# Ethnic inequalities in routes to diagnosis of cancer: a population-based UK cohort study

**DOI:** 10.1038/s41416-022-01847-x

**Published:** 2022-06-06

**Authors:** Tanimola Martins, Gary Abel, Obioha C. Ukoumunne, Luke T. A. Mounce, Sarah Price, Georgios Lyratzopoulos, Frank Chinegwundoh, William Hamilton

**Affiliations:** 1 College of Medicine and Health, University of Exeter, College House, St Luke’s Campus, Magdalen Road, Exeter, EX1 2LU UK; 2 National Institute for Health Research (NIHR) Applied Research Collaboration (ARC) South West Peninsula (PenARC), University of Exeter, Exeter, UK; 3 Epidemiology of Cancer Healthcare & Outcomes (ECHO) Group, University College London, 1-19 Torrington Place, London, WC1E 7HB UK; 4 Barts Health NHS Trust & Department of Health Sciences, University of London, London, UK

**Keywords:** Cancer epidemiology, Cancer epidemiology

## Abstract

**Background:**

UK Asian and Black ethnic groups have poorer outcomes for some cancers and are less likely to report a positive care experience than their White counterparts. This study investigated ethnic differences in the route to diagnosis (RTD) to identify areas in patients' cancer journeys where inequalities lie, and targeted intervention might have optimum impact.

**Methods:**

We analysed data of 243,825 patients with 10 cancers (2006–2016) from the RTD project linked to primary care data. Crude and adjusted proportions of patients diagnosed via six routes (emergency, elective GP referral, two-week wait (2WW), screen-detected, hospital, and Other routes) were calculated by ethnicity. Adjusted odds ratios (including two-way interactions between cancer and age, sex, IMD, and ethnicity) determined cancer-specific differences in RTD by ethnicity.

**Results:**

Across the 10 cancers studied, most patients were diagnosed via 2WW (36.4%), elective GP referral (23.2%), emergency (18.2%), hospital routes (10.3%), and screening (8.61%). Patients of Other ethnic group had the highest proportion of diagnosis via the emergency route, followed by White patients. Asian and Black group were more likely to be GP-referred, with the Black and Mixed groups also more likely to follow the 2WW route. However, there were notable cancer-specific differences in the RTD by ethnicity.

**Conclusion:**

Our findings suggest that, where inequalities exist, the adverse cancer outcomes among Asian and Black patients are unlikely to be arising solely from a poorer diagnostic process.

## Background

Tackling ethnic inequalities in cancer and other diseases is a public health priority, particularly in multi-ethnic societies like the UK and US, where disease outcomes vary significantly by ethnicity [1–5]. Cancer accounts for an estimated 166,000 and over 600,000 deaths per year in the UK and US, respectively [1, 5]. Historical data from the US shows that non-Hispanic Black Americans have higher mortality rates from nearly all cancers compared to other Americans [1]. In the UK, evidence suggests that Asian and Black women with breast cancer, and Black men with prostate cancer, have worse survival than other ethnic groups [2–4]. UK ethnic minority groups are also less likely to report a positive experience of care, including primary care and cancer care [6–9].

The causes of ethnic inequalities in cancer are complex and not fully understood. However, cancer survival rates are strongly associated with stage at diagnosis; early stage cancers have better chances of survival than those diagnosed at advanced stages [10, 11]. Advanced stage cancers may reflect one or more of several factors, including the tumour biology, uptake of screening, timeliness of medical help-seeking, intervals of diagnosis, and the presence of co-morbidities [12–14]. These factors may impact the route by which patients are diagnosed [15–17], although this relationship requires further investigation with respect to possible ethnic differences. In England, data on the route to diagnosis (RTD) of cancer patients are routinely gathered from several sources and reported publicly by the National Cancer Registration and Analysis Service (NCRAS) [18].

The RTD employs an algorithmic approach and describes patients’ care pathways to diagnosis of cancer as one of the eight routes (Box 1) [18]. It includes cancers detected via a screening programme, those reflecting the urgency of referral (emergency presentation, two-week wait (TWW), and elective GP referral), and cases where patients’ diagnostic journey started in secondary care (Outpatient elective or Other inpatients). The remaining two routes include cases identified based on death certificates and those with no useful record on RTD (unknowns) [18]. Earlier studies using RTD data examined cases identified up until 2013, with no consideration of possible variation by ethnicity [18]. Nonetheless, recent NCRAS estimates, updated with cases diagnosed up to 2016, showed differences in the proportions diagnosed via each route by ethnicity [19]. The analyses were mainly descriptive, with no consideration for the role of possible confounding factors; hence, the reported differences may be biased.

The NHS Long Term Plan includes a commitment to minimise inequalities, although it omitted any specific strategy to tackle ethnic differences in cancer diagnosis [20]. Exploring ethnic differences in the RTD may help to explain the observed ethnic variation in cancer outcomes and identify areas where targeted intervention might have optimum impact [18]. In the present study, we used primary care-linked patient records on cancers diagnosed between 2006 and 2016, focusing on ethnic differences in the RTD.

Box 1 The eight RTD used to categorise all tumours **Screen-Detected:** Detected via the breast, cervical or bowel screening programmes

**Two-Week Wait:** Urgent GP referral with a suspicion of cancer, using the two-week-wait (TWW) guidelines

**Elective GP Referral:** Routine and urgent referrals where the patient was not referred under the Two Week Wait referral route

**Other Outpatient:** An elective route starting with an outpatient appointment: either self-referral, consultant to consultant or other referral

**Inpatient Elective:** Where no earlier admission can be found prior to admission from a waiting list, booked or planned

**Emergency Presentation:** An emergency route via A&E, emergency GP referral, emergency transfer, emergency consultant outpatient referral or emergency admission or attendance

**Death Certificate Only:** No data available from Inpatient or outpatient Hospital Episode Statistics (HES), cancer waiting times (CWT), screening and with a death certificate only diagnosis flagged by the registry in the cancer analysis system

**Unknown:** No data available from inpatient or outpatient HES, CWT, screening within set time parameters or unknown referral

## Methods

### Study design and data sources

We conducted a population-based cohort study of patients diagnosed with one of the ten common cancers using data from the Clinical Practice Research Datalink (CPRD-Aurum) with linkage to Hospital Episode Statistics (HES), and the NCRAS cancer registry data. The CPRD-Aurum contains routinely gathered data from 890 consenting English practices (August 2019 release), with over 28 million patients eligible for linkage to other health care databases [21, 22]. It captures coded and anonymised data on patients’ medical history, including symptoms, investigations, diagnoses, prescriptions, referral, and demographics (e.g. age, gender, and ethnicity) [21, 22]. HES data—Admitted patient care and Outpatient elective—contains similar medical records on all hospital admissions and outpatient appointments, respectively, in England [21, 22]. The NCRAS cancer registry data includes records of all tumours diagnosed in England, alongside information about treatment, patient-reported outcomes, and the RTD.

### Participants

Eligible participants were aged at least 40 years at their index date (date at diagnosis), with an incident cancer recorded in cancer registry between 1 January 2006 and 31 December 2016. They had usable RTD and ethnicity records (Box 1 and Supplementary File 1), with at least one event recorded and 1 year of follow-up time in the CPRD prior to the index date to capture relevant information about primary care involvement in the diagnostic process. We excluded patients with cancers other than those listed below, and those diagnosed in an atypical sex (e.g. male breast/cervix). Furthermore, patients with missing ethnicity records in the CPRD and HES were excluded, as detailed below.

### Study variables

#### Cancer sites

Using the cancer registry data (considered the gold standard), we extracted patients records on 10 cancer sites: four most common UK non-skin cancers [lung (ICD10 C34), breast (C50), prostate (C61), colorectal (C18–C20)], and five sites commonly diagnosed in ethnic minority groups [oesophagus (C15), stomach (C16)), oral (C00–C14), cervix (C53), and myeloma (C90)], and ovarian cancer [2].

#### Route to diagnosis

Information on diagnostic routes was included in the NCRAS cancer registry data [22]. The methods used to assign diagnostic routes are detailed elsewhere [18, 22]. Briefly, all cancers diagnosed in England during the study period were assigned one of the eight diagnostic routes (Box 1), using data from the cancer registry, HES, Cancer Waiting Times, and National Health Service cancer screening programme. We excluded those with missing RTD data. For simplicity, Outpatient and Inpatient elective were merged into the hospital route, and those with unknown routes and Death Certificate Only (DCO) were merged into ‘Other’ routes.

#### Ethnicity

Information on patients’ ethnicity was derived from the CPRD and supplemented by HES ethnicity records, following the recommendations from previous studies [21, 23]. We extracted all ethnicity records from the CPRD using Medical codes, Read codes, and ethnicity-related terms in the CPRD Look-up files (Supplementary File 1). Identified ethnicities were reviewed by three researchers (TM, WH, and GA) to ascertain usable ethnicity codes, which were then collated into five major ethnic categories in line with the 2001 UK census groupings. These comprise: White (White British, White Irish, Any other White); Asian (Indian, Pakistani, Bangladeshi, Chinese, Other Asian); Black (Black Caribbean, Black African, Other Black); Mixed (White & Black Caribbean, White & Black African, White & Asian, Any other Mixed); and Other ethnic group.

For individuals with multiple ethnicity codes, we adapted an algorithm described by Mathur et al. to assign a single best ethnicity based on the most frequently—and most recently—recorded codes [24]. Supplementary File 2 shows the application of the algorithm to our data. For individuals with missing or unusable ethnicity codes in CPRD (including those recorded as unknown), we used ethnicity records in HES as substitutes. However, as previously reported [23], there was significant discordance between HES and CPRD ethnicity data, particularly for the Mixed and Other groups. For instance, in our data, there was 98% concordance for those coded White in both databases, 86% for those coded Black, and 85% for Asian. In contrast, 95% of those coded Mixed and 24% of those coded Other in the CPRD were coded White in HES. Nonetheless, we included both ethnic groups in analyses as they highlight important ethnic differences in RTD. Those listed as missing in both databases were excluded from analyses.

#### Other variables

Information on patient age, sex, level of deprivation, and multi-morbidities was identified from the CPRD. Age at diagnosis was calculated by subtracting year of birth from year of diagnosis, assigning a birthday of 1 July. We grouped age into four categories: 40–49, 50–59, 60–69, and ≥70 years. Deprivation was measured using quintiles of the 2015 Index of Multiple Deprivation (IMD) [25–27], available via CPRD linkage [22]. Here we employ groups based on national quintiles of the IMD [1–5] from least to most deprived. Data on morbidities were extracted from the CPRD using Medical codes relating to 37 long-term conditions as described by Cassell and colleagues [28]. For each patient, we derived a morbidity score as the sum of the General-outcome weighting assigned to each of their conditions, as described in Payne et al. [29]. Patients with none of these conditions were assigned a score of zero. The score was entered into the model as quartile-based groups of increasing morbidity burden.

### Statistical analysis

Analyses aimed to determine whether there are differences in the routes to diagnosis by ethnicity. We report the number and proportions (both crude and adjusted) of patients diagnosed via each route (emergency, elective GP referral, hospital, screening, TWW, and Other) by ethnicity. Adjusted proportions were predicted from multivariable multinomial logistic regression models, from which we computed differences in the adjusted percentage diagnosed via different routes between different ethnic groups. The adjusted proportions of RTD by ethnic group are the predicted proportions if the ethnic group had the same distribution on the confounding variables as observed in the data set for the entire sample (e.g. assuming that the proportion of the Black group that is female is the same as the proportion of the entire sample that is female). The primary outcome variable was RTD (reference route: TWW), with ethnicity as the main exposure (reference ethnicity: White). Other variables in the models were: age category, sex, IMD, morbidity scores, region, and cancer sites.

To check for differences in RTD across ethnic groups, we fitted several multivariable logistic regression models (including two-way interactions between cancer and age, sex, IMD, and ethnicity) to estimate cancer-specific odds ratios for individual RTD. This approach follows those employed in previous studies of RTD [30]. All analyses were carried out in Stata v16.1 (StataCorp, College Station, TX, USA) and the reporting guided by the STROBE framework for reporting observational studies [31]

## Results

### Participant characteristics

The overall cohort consisted of 297,803 patients, of whom 244,731 had usable ethnicity records for the ten cancer sites. After excluding those aged <40 years (*n* = 528) and those diagnosed in atypical sex—male breast (*n* = 377) and female prostate cancer (*n* = 1)—243,825 records were available for analysis. Table 1 shows the characteristics of the 243,825 patients. In all, 151,163 (62.0%) were of White ethnic background, 4611 (1.89%) Black, 4479 (1.84%) Asian, 79,609 (32.7%) Mixed, and 3963 (1.63%) were of Other ethnic background. Compared to the 2011 national census figures for England and Wales [32, 33], males were overrepresented in the Black group and females were overrepresented in the Asian and Other ethnic groups in our sample. In that census, 48% of the Black group were males, and 50% of Asian and 45% of the Other group were females—the proportion in our sample was 62, 54, and 49%, respectively. Consistent with the population distribution [33], at diagnosis, Black and Asian patients were younger, lived in more deprived areas, and were mostly concentrated in London compared to White patients, who were unevenly distributed across 10 English regions. The Mixed group was similar to the White group in terms of age, sex, and level of deprivation, though had more morbidity than the former. The Other group was older but with less morbidity than the White group.

**Table 1:** Participants characteristics by ethnicity.

	All *N* = 243,825	White—*n* (%) 151,163 (62.0)	Black—*n* (%) 4611 (1.89)	Asian—*n* (%) 4479 (1.84)	Mixed—*n* (%) 79,609 (32.7)	Other—*n* (%) 3963 (1.63)
Gender						
Female	120,289 (49.3)	74,643 (49.4)	1748 (37.9)	2402 (53.6)	39,485 (49.3)	2011 (50.7)
Male	123,536 (50.7)	76,520 (50.6)	2863 (62.1)	2077 (46.4)	40,124 (50.4)	1952 (49.3)
Age, years						
Median (IQR) years	70 (62–79)	71 (62–80)	66 (54–75)	64 (54–74)	71 (62–79)	72 (62–82)
40–49	17,171 (7.04)	10,414 (6.89)	705 (15.3)	696 (15.5)	5085 (6.39)	271 (6.84)
50–59	35,761 (14.7)	21,870 (14.5)	1076 (23.3)	1040 (23.2)	11,177 (14.0)	598 (15.1)
60–69	66,293 (27.2)	40,883 (27.1)	1008 (21.9)	1178 (26.3)	22,271 (27.9)	953 (24.1)
≥70	124,600 (51.1)	77,996 (51.6)	1822 (39.5)	1565 (34.9)	41,076 (51.6)	2141 (54.0)
IMD^a^						
1	57,516 (23.6)	37,740 (24.9)	196 (4.25)	723 (16.2)	17,868 (22.5)	989 (24.9)
2	53,170 (21.8)	33,496 (22.2)	294 (6.38)	740 (16.5)	17,730 (22.8)	910 (22.9)
3	48,424 (19.9)	30,034 (19.9)	776 (16.8)	949 (21.2)	15,897 (19.9)	768 (19.4)
4	43,616 (17.9)	25,836 (17.1)	1328 (28.8)	1010 (22.6)	14,773 (18.6)	669 (16.9)
5	40,979 (16.8)	23,975 (15.9)	2015 (43.7)	1055 (23.6)	13,309 (16.7)	625 (15.8)
Morbidity scores						
0	26,936 (11.1)	18,394 (12.2)	608 (13.2)	591 (13.2)	6556 (8.24)	787 (19.9)
1	45,596 (18.7)	29,085 (19.2)	860 (18.7)	780 (17.4)	14,042 (17.6)	829 (20.9)
2	54,589 (22.4)	33,806 (22.4)	1132 (24.6)	1043 (23.3)	17,627 (22.1)	981 (24.8)
3	56,809 (23.3)	34,267 (22.7)	1057 (22.9)	1137 (25.4)	19,535 (24.5)	813 (20.5)
4	59,895 (24.6)	35,611 (23.6)	954 (20.7)	928 (20.7)	21,849 (27.5)	553 (13.9)
Region^a^						
North East	12,105 (4.97)	6559 (4.34)	16 (0.35)	51 (1.14)	5429 (6.82)	50 (1.26)
North West	44,004 (18.1)	28,946 (19.2)	384 (8.35)	486 (10.9)	13,522 (16.9)	666 (16.8)
Yorkshire	9731 (3.99)	5432 (3.59)	24 (0.52)	67 (1.50)	4074 (5.12)	134 (3.38)
East Midlands	5169 (2.12)	2813 (1.86)	53 (1.15)	91 (2.04)	2151 (2.70)	61 (1.54)
West Midlands	47,863 (19.6)	29,387 (19.4)	674 (14.7)	986 (22.1)	16,208 (20.4)	608 (15.4)
East of England	12,869 (5.28)	8943 (5.92)	79 (1.72)	166 (3.71)	3433 (4.31)	248 (6.26)
South West	36,476 (14.9)	20,570 (13.6)	180 (3.91)	153 (3.42)	15,051 (18.9)	522 (13.2)
South Central	27,693 (11.4)	17,963 (11.9)	157 (3.41)	320 (7.16)	8640 (10.9)	613 (15.5)
London	27,106 (11.1)	15,148 (10.0)	2940 (63.9)	1871 (41.9)	6669 (8.38)	478 (12.1)
South East	20,761 (8.52)	15,384 (10.2)	93 (2.02)	279 (6.24)	4424 (5.56)	581 (14.7)
Cancer site						
Breast	57,056 (23.4)	34,409 (22.8)	956 (20.7)	1410 (31.5)	19,538 (24.5)	743 (18.7)
Lung	48,294 (19.8)	31,220 (20.7)	498 (10.8)	602 (13.4)	14,907 (18.7)	1067 (26.9)
Prostate	51,492 (21.1)	30,743 (20.3)	1780 (38.6)	796 (17.8)	17,600 (22.1)	573 (14.5)
Colorectal	45,257 (18.6)	28,334 (18.7)	604 (13.1)	732 (16.3)	14,781 (18.6)	806 (20.3)
Oesophagus	9697 (3.98)	6,291 (4.16)	69 (1.50)	108 (2.41)	3018 (3.19)	211 (5.32)
Stomach	8066 (3.31)	5058 (3.35)	211 (4.58)	158 (3.53)	2425 (3.05)	214 (5.40)
Oral	8541 (3.50)	5427 (3.59)	96 (2.08)	262 (5.85)	2670 (3.35)	86 (2.17)
Ovary	7565 (3.10)	4849 (3.21)	77 (1.67)	197 (4.40)	2302 (2.89)	140 (3.53)
Myeloma	5843 (2.40)	3544 (2.34)	271 (5.88)	162 (3.62)	1778 (2.23)	88 (2.22)
Cervix	2014 (0.83)	1,288 (0.85)	49 (1.06)	52 (1.16)	590 (0.74)	35 (0.88)

^a^Missing record of IMD (*n* = 120 (0.05%)) and Region (*n* = 48 (0.02%)).

### Cancer sites

Four-fifths of our sample were diagnosed with breast, lung, prostate, or colorectal cancer although the proportion diagnosed with each cancer type differed by ethnicity. Patients of Other ethnic background had the highest proportion with lung, colorectal, oesophagus, or stomach cancer. The Black group had the highest proportions with myeloma, prostate, or stomach cancer, while the Asian group had the highest proportion diagnosed with breast, cervical, ovarian, or oral cancer. Patients of White ethnic background had the second-highest proportion with lung, colorectal, or oesophageal cancer, whereas the Mixed group had the second-highest proportion with breast or prostate cancer (Table 1).

### Ethnic differences in diagnostic routes

**Fig. 1:**
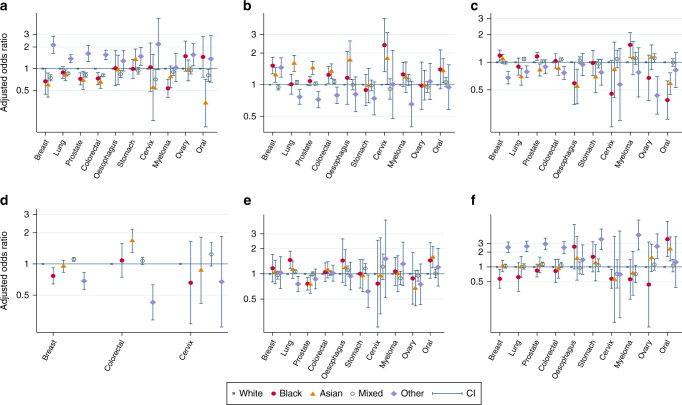
Cancer-specific adjusted odds ratios and 95% confidence intervals for individual RTD. **a** Emergency presentation; **b** Elective GP referral; **c** Two-week wait; **d** Screen-detected; **e** Hospital routes; and **f** Other routes.

For all 10 cancers combined, 36.4% (*n* = 88,615) followed the TWW route, 23.2% (*n* = 56,580) were diagnosed following elective GP referral, 18.2% (*n* = 44,431) presented as emergencies, 10.3% (*n* = 24,983) via the hospital routes, 8.61% (*n* = 20,970) were screen-detected, and 3.32% (*n* = 8078) were diagnosed via other routes. There were substantial differences by ethnicity in the RTD (Table 2 and Supplementary File 3) and strong evidence (*p* < 0.01) that these differences varied by cancer site (Fig. 1).

**Table 2:** Number and adjusted proportions of RTD by ethnicity.

RTD	White (*n*)	Black (*n*)	Asian (*n*)	Mixed (*n*)	Other (*n*)	Total (*n*)
Emergency	28,963	680	606	13,070	1112	44,431 18.2
Crude %	19.2	14.8	13.6	16.4	28.1	
Adjusted % (95% CI)	18.9 (18.8–19.1)	16.1 (15.1–17.2)	15.6 (14.5–16.6)	16.7 (16.5–17.0)	24.9 (23.7–26.1)	
*p* value	—	<0.001	<0.001	<0.001	<0.001	
Elective GP-referral	34,680	1428	1273	18,428	771	56,580 23.2
Crude %	22.9	31.1	28.5	23.2	19.5	
Adjusted % (95% CI)	22.9 (22.8–23.2)	25.6 (24.4–26.9)	28.3 (27.0–29.6)	23.4 (23.1–23.7)	20.5 (19.2–21.7)	
*p* value	—	<0.001		0.01	<0.001	
Hospital	15,539	453	438	8174	379	24,983 10.3
Crude %	10.3	9.85	9.80	10.3	9.57	
Adjusted % (95% CI)	10. 2(10.0–10.3)	10.3 (9.40–11.2)	10.6 (9.60–11.5)	10.4 (10.2–10.6)	9.20 (8.30–10.1)	
*p* value	—	0.85	0.45	0.07	0.03	
Screen	12,500	235	487	7551	197	20,970 8.61
Crude %	8.27	5.11	10.9	9.49	4.98	
Adjusted % (95% CI)	8.46 (8.35–8.58)	7.35 (6.59–8.11)	8.70 (8.09–9.31)	9.00 (8.84–9.16)	6.25 (5.54–6.97)	
*p* value	—	0.004	0.46	<0.001	<0.001	
TWW	54,473	1683	1494	29,798	1167	88,615 36.4
Crude %	36.1	36.6	33.4	37.5	29.5	
Adjusted % (95% CI)	36.2 (36.0–36.5)	38.0 (36.6–39.5)	33.4 (31.9–34.7)	37.0 (36.7–37.4)	31.3 (29.9–32.7)	
*p* value	—	0.02	<0.001	<0.001	<0.001	
Other	4908	119	170	2548	333	8078 3.32
Crude %	3.25	2.59	3.80	3.20	8.41	
Adjusted % (95% CI)	3.18 (3.09–3.27)	2.56 (2.10–3.03)	3.50 (2.98–4.02)	3.38 (3.25–3.51)	7.84 (7.04–8.65)	
*p* value	—	0.01	0.24	0.01	<0.001	
Total (n)	151,063	4598	4468	79,569	3959	243,657

Adjusted for age category, sex, IMD, morbidity scores, region, and cancer sites. The ‘Other’ RTD comprise cancers diagnosed via Death Certificate Only and those with unknown RTD.

### Ethnic differences in emergency diagnosis

Crude estimates (Table 2) showed that greater proportions of patients of Other ethnic group [28.1% (*n* = 1112)], followed by the White group [19.2% (*n* = 28,963)] were diagnosed as emergencies compared with Mixed [16.4% (*n* = 13,070)], Asian [13.6% (*n* = 606)], or Black [14.8% (*n* = 680)] patients. These differences held across all patients’ characteristics (age groups, sex, quintiles of deprivation, morbidity scores, and regions) but differed by cancer types (Supplementary File 3a). Figure 1a shows site-specific adjusted odds ratio (and confidence intervals) of the differences in emergency diagnosis by ethnicity (additional detail in Supplementary File 4). Specifically, compared with the White group, the odds of emergency diagnosis were significantly higher among the Other ethnic group with breast, lung, prostate, colorectal, stomach, or ovarian cancer, but similar for the remaining sites. In contrast, Asian patients (with breast, lung, prostate, colorectal, or oral), plus Black patients (with prostate, colorectal, or myeloma), and patients of Mixed ethnic background (with breast, lung, prostate, colorectal, oesophageal, cervical or oral cancer) all have significantly lower odds of emergency diagnosis than White patients. We found no difference between the Black and White groups in emergency diagnosis of lung, oesophageal, stomach, cervix, ovarian, or oral cancers; likewise, there was no difference between Asian and White patients in emergency diagnosis of myeloma, ovarian, cervical, stomach, or oesophageal cancer. Furthermore, there was no difference in the odds of emergency diagnosis between the White and Mixed groups with stomach, myeloma, or ovarian cancer. However, after adjusting for age, gender, IMD, morbidity scores, region, and cancer site, the proportion of patients diagnosed as emergencies remained significantly higher for the Other ethnic group but lower for the Asian, Black, and Mixed group than the White group (Table 2).

### Ethnic differences in elective GP-referral routes

A greater proportion of Black [31.1% (*n* = 1428)] and Asian [28.5% (*n* = 1273)] compared to the Mixed [23.2% (*n* = 18,428)], White [22.9% (*n* = 34,680)], and Other group [19.5% (*n* = 771)] were diagnosed via elective GP referral (Table 2 and Supplementary File 3b). This pattern was observed across all patients’ characteristics but differed slightly by cancer types (Fig. 1b). Compared with the White group, the odds of diagnosis via elective GP referral routes were higher in the Other group with breast cancer, Black group (with breast, colorectal or cervical), Asian group (with lung, breast, prostate, colorectal, cervix, oral or oesophageal), and the Mixed group with lung or colorectal cancer. For three sites (lung, prostate, and colorectal), the odds of diagnosis via this route were lower in the Other group than the White group. For stomach, myeloma, and ovarian cancer, we found no difference by ethnicity. However, after adjusting for other variables, the proportion of White patients diagnosed via elective GP referral overall remained lower than Asian, Black, and Mixed group but higher than the Other group.

### Ethnic differences in TWW route

Both crude and adjusted estimates showed that fewer patients of Asian and Other groups than the White, Black, or Mixed group were diagnosed via TWW route (Table 2), with adjusted proportions as follows: Other [31.3% (*n* = 1167)], Asian [33.4% (*n* = 1494)], White [36.2% (*n* = 54,473)], Mixed [37% (*n* = 29,798)], and Black [38% (*n* = 1683)]. However, subgroup analyses by cancer revealed more heterogeneity among those diagnosed via this route (Supplementary File 3e and Fig. 1c). For instance, we found significantly higher odds of diagnosis via TWW route in the Black group (with breast, prostate, or myeloma) and Mixed group (with lung or colorectal cancer) than in the White group. By contrast, Black group (with oesophageal or oral), Asian (with lung, prostate, oesophageal or oral), and the Other group (with breast, lung, colorectal, or ovarian cancer) all had lower odds of diagnosis via the TWW route. For stomach and cervical cancer, we found no difference by ethnicity in diagnosis via TWW route.

### Ethnic differences in screen-detected route

Fewer patients of Other [4.98% (*n* = 197)] and Black [5.11% (*n* = 235)] groups were diagnosed via screening compared to White [8.27% (*n* = 12,500)], Asian [10.9% (*n* = 487)], or Mixed [9.49% (*n* = 7551)] patients. The proportion of Black and Other groups diagnosed via screening was lower, particularly for breast, and for colorectal cancer in the Other group (Supplementary File 3d and Fig. 1d). For colorectal and breast cancers, Asian and Mixed groups, respectively, had greater odds of diagnosis via screening than other ethnicities. We found no evidence of ethnic differences in screen-detected cervical cancer (Fig. 1d). Adjusted proportions showed that the Other and Black groups were, on the whole, less likely than the Mixed, Asian, and White groups to be diagnosed via screening.

### Ethnic differences in diagnosis via Hospital route

Both crude and adjusted estimates revealed little difference by ethnicity in the proportion diagnosed via the hospital routes (Table 2), with adjusted proportions as follows: White [10.2% (*n* = 15,539)], Black [10.3% (*n* = 453)], Asian [10.6% (*n* = 438)], Mixed [10.4% (*n* = 8174)], and Other [9.2% (*n* = 379)]. However, subgroup analyses (Fig. 1e and Supplementary File 3c) showed greater odds of diagnosis via the hospital route than the White group for lung (Black), oral (Asian), and stomach (Mixed) cancers. Conversely, the odds of diagnosis via the hospital route were lower compared to the White group only for the Asian group, the Black group with prostate, and the Other group with lung and stomach cancer.

### Ethnic differences in diagnosis via Other routes

The proportion diagnosed via the Other routes were similar by ethnicity, with the exception of the Other ethnic group, with adjusted proportion as follows: White [3.18% (*n* = 4908)], Black [2.56% (*n* = 119)], Asian [3.5% (*n* = 170)], Mixed [3.38% (*n* = 2548)], and Other group [7.84% (*n* = 333)]. Subgroup analyses revealed the same trend, with the Other ethnic group having greater odds of diagnosis via the other routes in seven sites (breast, lung, prostate, colorectal, stomach, myeloma, and ovarian cancer) compared to the White group. We found greater odds of diagnosis via other routes in Asian and Black group with oral cancer, with a reverse of the same in the Black group with breast and cervical cancer. For other cancer types, we found no evidence of ethnic difference among those diagnosed via other routes (Fig. 1f and Supplementary File 3f).

## Discussion

This study identified differences by ethnicity in the RTD of ten common cancers. We found that patients of Other ethnic background were most likely to be diagnosed via the emergency or other routes and were least likely to be screen-detected or follow the TWW route compared with patients of other ethnic backgrounds. Patients of White background were more likely than those of Asian, Black, or Mixed ethnic backgrounds to be diagnosed via the emergency route. Being Black increased the probability of diagnosis via elective GP referral or TWW routes but reduced the chances of cancer diagnosis via screening compared to being White. Conversely, being Asian increased the probability of diagnosis via elective GP referral but was also linked to reduced chances of TWW referral. Patients of Mixed ethnic background had a greater likelihood of diagnosis via screening, elective GP referral, and TWW routes compared with someone of White ethnic background.

### Strengths and limitations

In a relatively under-researched subject area, this study is the first to investigate ethnic differences in RTD of cancer, using primary care data linked to hospital and cancer registry data. It had a large sample size and examined ten cancers, including the four most common, plus six sites frequently diagnosed in the UK ethnic minority population. We used robust methods to identify variables included in our analyses. For instance, data on morbidities were extracted from the CPRD, with the Cambridge Multimorbidity Score assigned using validated methodology that outperforms alternatives such as the Charlson Index [29]. Gold standard information on cancer sites and RTD were obtained from NCRAS cancer registry data. The RTD data derivation methods are transparent [18], and the database is widely accepted as the strongest available source of routes to diagnosis. While diagnostic route in some patients will inevitably have been misclassified, this is highly unlikely to have occurred sufficiently frequently to affect our results materially. RTD data combines routine and urgent GP-referral routes as *GP-referral*, here labelled ‘elective GP’ as distinct from TWW referral. Therefore, differences by ethnicity in the proportion diagnosed via either of these subroutes could not be ascertained.

Data on patients’ ethnicity—defined in line with UK national census groupings—were identified from the CPRD and HES, with 99% completeness. Priority was accorded to ethnicity records in the CPRD over HES, in line with previous recommendations [23, 34]. We used combined ethnic groupings for simplicity and to maximise our power, recognising that this hides differences within combined groups. For instance, we categorised the Mixed—White and Asian, Mixed—White and Black, and Other Mixed groups (including the so-called ‘British or British Mixed’) as Mixed. It is conceivable that some of the British or British Mixed group, in particular, are White, although this was not apparent from our data set with the majority from this group having no substitute ethnicity code. The alternative was to use the 16 ethnic subgroups in the 2001 census, which would have reduced power (particularly in rarer cancers) and made interpretation of our findings unwieldy.

Our cohort was limited to patients with a recorded event during the study period—and 1-year follow-up time in the CPRD before the index date—to capture data on relevant covariates in this study. This restriction may have introduced bias to our study, as it precludes a small number of patients whose first medical contact is in secondary care.

### Interpretation of findings

Reflecting the demography of major ethnic minorities in the UK, Asian and Black patients in this study were younger, lived in more deprived areas, and were concentrated in London compared to White patients [35, 36]. We found ethnic differences in cancer diagnosis consistent with previous reports [2, 37] although our finding of a higher proportion of female breast cancer among Asian patients is new and warrants further exploration.

Around 18% of our sample presented as emergencies; similar to recent estimates for all malignant neoplasms diagnosed between 2006 and 2016 [19], but lower than the proportion reported in previous studies [18, 38]. We found significant ethnic differences in the proportion diagnosed via emergency presentation, including differences by cancer types, with the Other ethnic group, followed by the White group more likely than other ethnicities to follow this route. This finding must be interpreted with caution considering the heterogeneity within the Other group (including Arab, those with unknown and several other uncategorised ethnicities), with no previous UK studies specifically exploring cancer inequalities in this group. Around 25% of the Other group in our sample were diagnosed as emergencies, similar to the national figure in the 2010s [18]. The Other group had the smallest proportion diagnosed following elective GP, screening or TWW referral, and the highest proportion diagnosed via DCO or Unknown routes. However, patients in this group were slightly older and had the highest proportions of lung, colorectal, oesophagus or stomach cancers, which are associated with increased risk of emergency presentation [18, 38]. It is possible that the Other group over-represents patients with non-usual UK residence, whose medical history or clinical encounters with the NHS, and by extension cancer pathways, may differ to those usually residing in the UK [39]. Further research will need to unpick this ethnic group to fully understand the complex factors contributing to their higher risks of emergency diagnosis.

Our finding that the White group was more likely to follow the emergency diagnosis route than Asian and Black groups was unexpected, given known ethnic inequalities in cancer outcomes [4, 40]. In addition, previous studies showed that Asian and Black groups have a greater frequency of multiple pre-referral consultations, [41, 42], which in reality, would augur a higher proportion of emergency diagnoses in both groups than the White group. However, we observed that patients from these groups were more likely than their White counterparts to be GP-referred either electively or via the TWW route (mostly for Black patients). These findings are consistent with reported greater use of primary care among Asians and Black patients [42, 43] and may account for the differences in emergency diagnosis observed here. For oral cancer, we found evidence that Asians and Black patients were more likely than White patients to be diagnosed via DCO or Unknown routes. It is possible some of these were referred by the dentist, not coded in medical records, or missed completely.

Overall, Black patients were more likely than patients from other ethnicities to be diagnosed via the TWW route, although this was not the case for seven of the ten cancers in this study, for which we found no difference or lower odds of TWW referral for Black patients. Asian patients were less likely than Black, Mixed, and White patients to be diagnosed via the TWW route. These differences in TWW by cancer types may reflect variation in specialist referral threshold [44, 45], with GPs setting higher thresholds, particularly for Asian and Black patients, than their White counterparts. We recently showed that in patients with lower urinary tract symptoms, GPs were selective in offering investigation based on patient ethnicity, although this was partly due to the presence of co-morbidities [46]. This may be the case for other cancer symptoms, with the potential to impact on referral to the specialist, in this case via TWW.

However, our findings may reflect specific characteristics of the general practices that Asian and Black patients use, for example practices with higher referral thresholds and access to diagnostic and specialist services [47]. Such clustering of ethnic minorities has been shown to explain around half of the disparity between White and Asian patients in terms of patient care experience [7]. Indeed our findings are consistent with previous evidence indicating that practices with a greater percentage of ethnic minorities (particularly the South-Asian group) were associated with lower use of the TWW pathway [48]. Previous work also shows substantial variation between practices in the propensity to refer patients for specialist care, which may be attributed to the wider health economy within which practices operate [49]. Further work would be necessary to unpick the extent to which these systemic factors impact ethnic minority patients’ cancer journey.

Black patients in our sample were less likely to be diagnosed via screening, consistent with their lower uptake of screening opportunities [50, 51]. More targeted efforts might be required to change this trend. As Black patients frequently use primary care [41], this may provide the ideal setting for targeted interventions to improve the awareness and uptake of screening [52]. A recent trial showed that targeted intervention to promote cancer awareness and help seeking was associated with improved consultation rates [53]. Such intervention may be adapted to promote breast cancer screening uptake in Black women.

The RTD in the Mixed group are mostly more favourable than in the White group.

## Conclusions

This study sought to identify a possible explanation for poorer cancer outcomes among ethnic minority groups in the UK, particularly the Asian and Black groups. Against our original hypothesis, emergency route to diagnosis—which might be a marker of poorer diagnostic services—was more common among the White population than in Asian and Black groups, although this was not found in all cancer sites. This suggests that inequalities in symptomatic diagnosis of cancer, especially breast cancer, are unlikely to be a major cause of ethnic inequalities in cancer outcomes in the UK. This is supported by our findings of a greater proportion of Black and Asian patients diagnosed via elective GP referral, and more Black patients diagnosed via TWW route. However, patients of Other ethnic backgrounds have the lowest proportion diagnosed via screening, elective GP and TWW referral, with the highest proportion presenting as emergencies. Further research is necessary to fully understand the situation with patients from this complex group alongside those of Mixed ethnic group. There is a need for more robust methods of collecting ethnicity data to fully understand the extent and causes of ethnic inequalities in cancer and other diseases. Ideally, ethnicity groupings within the health service should align with those of the Office for National Statistics for consistency. Authorities should consider dropping unhelpful labels such as the British or British Mixed group, which poorly defines patients' ethnicity.

## Supplementary information

Reproducibility checklist

Supplementary File 1

Supplementary File 2 - revised

Supplementary file 3

Supplementary file 4

Supplementary File 5
