# Supplementary material for: Ethnic inequalities in routes to diagnosis of cancer: a population-based UK cohort study
Source: Br J Cancer. 2022 Jun 6;127(5):863–71. doi: 10.1038/s41416-022-01847-x (PMC9427836; doi:10.1038/s41416-022-01847-x)
Supplement: Supplementary file 2 — Supplementary File 1 [file 41416_2022_1847_MOESM2_ESM.docx]

**Supplementary File 1: Ethnicity related codes and terms in the CPRD**

| **Medcodeid** | **Originalreadcode** | **Cleansedreadcode** | **Term** | **Ethnicity** |
| --- | --- | --- | --- | --- |
| 1063981000000110 | 9i00 | 9i00.00 | White British - ethnic category 2001 census | White |
| 1564601000006110 | EMISNQCZ2 |  | Czech | White |
| 411594011 | 9SAA-1 | 9SAA.11 | Greek (NMO) | White |
| 196641000006110 | 134B | 134B.00 | RACE: Caucasian | White |
| 138181000000111 | 9i2D | 9i2D.00 | Traveller - ethnic category 2001 census | White |
| 1968081000006110 | 9t03 | 9t03.00 | White: other White backgrd- Eng+Wales ethnic cat 2011 census | White |
| 1573201000006110 | EMISNQPO8 |  | Portuguese | White |
| 1968051000006110 | 9t00 | 9t00.00 | White:Eng/Welsh/Scot/NI/Brit - England and Wales 2011 census | White |
| 2615461000000110 | 9TC1 |  | Czech Roma | White |
| 937391000006115 | 9i2R | 9i2R.00 | Oth White European/European unsp/Mixed European 2001 census | White |
| 937301000006110 | 9i2G | 9i2G.00 | Baltic Estonian/Latvian/Lithuanian - ethn categ 2001 census | White |
| 253629014 | 2261 | 2261 | O/E - Europeanoid | White |
| 138201000000110 | 9i2F | 9i2F.00 | Polish - ethnic category 2001 census | White |
| 2487321000000110 | 9t21 | 9t21.00 | White: other British - Scotland ethnic category 2011 census | White |
| 1968251000006110 | 9t11 | 9t11.00 | Irish Traveller - Northern Ireland ethnic cat 2011 census | White |
| 141311000000112 | 9i2 | 9i2..00 | Other White background - ethnic category 2001 census | White |
| 286007012 | 9T11 | 9T11.00 | New Zealand European | White |
| 158481000000115 | 9i2B | 9i2B.00 | Italian - ethnic category 2001 census | White |
| 250222012 | 1341 | 1341 | European origin | White |
| 1160331000000110 | 9T6 | 9T6..00 | Czech | White |
| 459727011 | 9S11 | 9S11.00 | White Irish | White |
| 1551471000000110 | 9T7 | 9T7..00 | Slovak | White |
| 141301000000110 | 9i1 | 9i1..00 | Irish - ethnic category 2001 census | White |
| 1780408016 | 9S14 | 9S14.00 | Other white British ethnic group | White |
| 138171000000114 | 9i2C | 9i2C.00 | Irish Traveller - ethnic category 2001 census | White |
| 141661000000115 | 9i26 | 9i26.00 | Cypriot (part not stated) - ethnic category 2001 census | White |
| 285978014 | 9SA9 | 9SA9.00 | Irish (NMO) | White |
| 937371000006116 | 9i2P | 9i2P.00 | Other republics former Yugoslavia - ethnic categ 2001 census | White |
| 142751000000115 | 9i2L | 9i2L.00 | Bosnian - ethnic category 2001 census | White |
| 1968071000006110 | 9t02 | 9t02.00 | White: Gypsy/Irish Traveller - Eng+Wales eth cat 2011 census | White |
| 2971841000006110 | ^ESCTBU297184 |  | Bulgarian | White |
| 459726019 | 9S10 | 9S10.00 | White British | White |
| 1565511000006110 | EMISNQPO6 |  | Portuguese | White |
| 285987017 | 9SAC | 9SAC.00 | Other European (NMO) | White |
| 138191000000113 | 9i2E | 9i2E.00 | Gypsy/Romany - ethnic category 2001 census | White |
| 157281000000117 | 9i20 | 9i20.00 | English - ethnic category 2001 census | White |
| 405070017 | 9SAA | 9SAA.00 | Greek/Greek Cypriot (NMO) | White |
| 1064041000000110 | 9i10 | 9i10.00 | White Irish - ethnic category 2001 census | White |
| 459728018 | 9S12 | 9S12.00 | Other white ethnic group | White |
| 2537217015 | 134N | 134N.00 | Race: White | White |
| 141431000000111 | 9i21 | 9i21.00 | Scottish - ethnic category 2001 census | White |
| 286009010 | 9T12 | 9T12.00 | Other European in New Zealand | White |
| 1780407014 | 9S13 | 9S13.00 | White Scottish | White |
| 142691000000116 | 9i25 | 9i25.00 | Ulster Scots - ethnic category 2001 census | White |
| 141441000000119 | 9i22 | 9i22.00 | Welsh - ethnic category 2001 census | White |
| 142741000000118 | 9i2J | 9i2J.00 | Kosovan - ethnic category 2001 census | White |
| 138241000000113 | 9iF6 | 9iF6.00 | Jewish - ethnic category 2001 census | White |
| 2487361000000110 | 9t22 | 9t22.00 | White: Irish - Scotland ethnic category 2011 census | White |
| 1968441000006110 | 9t23 | 9t23.00 | White: Gypsy/Irish Traveller - Scotland ethnic cat 2011 cens | White |
| 411595012 | 9SAA-2 | 9SAA.12 | Greek Cypriot (NMO) | White |
| 141451000000116 | 9i24 | 9i24.00 | Northern Irish - ethnic category 2001 census | White |
| 6846371000006110 | ^ESCTCA684637 |  | Caucasian | White |
| 156921000000110 | 9i29 | 9i29.00 | Turkish - ethnic category 2001 census | White |
| 285925010 | 9S1 | 9S1..00 | White | White |
| 142701000000116 | 9i27 | 9i27.00 | Greek - ethnic category 2001 census | White |
| 2487481000000110 | 9t24 | 9t24.00 | White: Polish - Scotland ethnic category 2011 census | White |
| 1968461000006110 | 9t25 | 9t25.00 | White: other White ethnic grp- Scotland ethnic cat 2011 cens | White |
| 2487281000000110 | 9t20 | 9t20.00 | White: Scottish - Scotland ethnic category 2011 census | White |
| 459786016 | 9SI | 9SI..00 | Irish traveller | White |
| 1565671000006110 | EMISNQSL1 |  | Slovak | White |
| 937411000006115 | 9i2T | 9i2T.00 | Other White or White unspecified ethnic category 2001 census | White |
| 1158301000000110 | 9T5 | 9T5..00 | Bulgarian | White |
| 141461000000118 | 9i23 | 9i23.00 | Cornish - ethnic category 2001 census | White |
| 133078012 | 9T8 | 9T8..00 | Portuguese | White |
| 2484671000000110 | 9t01 | 9t01.00 | White: Irish - England and Wales ethnic category 2011 census | White |
| 157991000000110 | 9i2N | 9i2N.00 | Serbian - ethnic category 2001 census | White |
| 1564411000006110 | EMISNQBU2 |  | Bulgarian | White |
| 142761000000117 | 9i2M | 9i2M.00 | Croatian - ethnic category 2001 census | White |
| 138231000000116 | 9i2K | 9i2K.00 | Albanian - ethnic category 2001 census | White |
| 142781000000114 | 9i2Q | 9i2Q.00 | Mixed Irish and other White - ethnic category 2001 census | White |
| 2486161000000110 | 9t10 | 9t10.00 | White - Northern Ireland ethnic category 2011 census | White |
| 142711000000119 | 9i28 | 9i28.00 | Greek Cypriot - ethnic category 2001 census | White |
| 1968201000006110 | 9t0F | 9t0F.00 | Black/Afr/Carib/Black Brit: other Black- Eng+Wales 2011 cens | Black |
| 285953010 | 9S52 | 9S52.00 | Other Black - Black/Asian orig | Black |
| 411576017 | 9S45-1 | 9S45.11 | Black East African Asian | Black |
| 405064011 | 9S42 | 9S42.00 | Black Caribbean/W.I./Guyana | Black |
| 141391000000115 | 9iC | 9iC..00 | African - ethnic category 2001 census | Black |
| 405067016 | 9SA3 | 9SA3.00 | Caribbean I./W.I./Guyana (NMO) | Black |
| 1968191000006110 | 9t0E | 9t0E.00 | Black/African/Caribbn/Black Brit: Caribbean - Eng+Wales 2011 | Black |
| 411574019 | 9S43-2 | 9S43.12 | Black Arab | Black |
| 285943014 | 9S44 | 9S44.00 | Black - other African country | Black |
| 141601000000119 | 9iD1 | 9iD1.00 | Nigerian - ethnic category 2001 census | Black |
| 141571000000114 | 9iA7 | 9iA7.00 | Caribbean Asian - ethnic category 2001 census | Black |
| 453109012 | 9S42-2 | 9S42.12 | Black West Indian | Black |
| 1968351000006110 | 9t1B | 9t1B.00 | Black/Afri/Carib/Black Brit: African- NI eth cat 2011 census | Black |
| 285951012 | 9S5 | 9S5..00 | Black - other, mixed | Black |
| 30683015 | 9S3 | 9S3..00 | Black African | Black |
| 285950013 | 9S48 | 9S48.00 | Black Black - other | Black |
| 459731017 | ESCTBL5-1 |  | Black | Black |
| 459782019 | 9SG | 9SG..00 | Other black ethnic group | Black |
| 141591000000113 | 9iD0 | 9iD0.00 | Somali - ethnic category 2001 census | Black |
| 514611000006111 | 9S2 | 9S2..00 | Black Caribbean | Black |
| 1968541000006110 | 9t2D | 9t2D.00 | African: any other African - Scotland ethnic cat 2011 census | Black |
| 4916941000006110 | ^ESCTAF491694 |  | African country | Black |
| 1968551000006110 | 9t2E | 9t2E.00 | Carib/Black: Caribbean/Carib Scot/Carib Brit- Scotland 2011 | Black |
| 411580010 | 9SA3-3 | 9SA3.13 | Guyana (NMO) | Black |
| 459872011 | 13g | 13g..00 | Country of birth (African) | Black |
| 253630016 | 2262 | 2262 | O/E - Negroid | Black |
| 411575018 | 9S43-3 | 9S43.13 | Black Iranian | Black |
| 1968371000006110 | 9t1D | 9t1D.00 | Black/Afri/Carib/Black Brit: other - NI eth cat 2011 census | Black |
| 411579012 | 9SA3-2 | 9SA3.12 | West Indian (NMO) | Black |
| 250223019 | 1342 | 1342 | African origin | Black |
| 1968531000006110 | 9t2C | 9t2C.00 | African: African/African Scot/African Brit - Scotland 2011 | Black |
| 459915019 | 13g9 | 13g9.00 | Born in Central African Republic | Black |
| 158351000000119 | 9iD | 9iD..00 | Other Black background - ethnic category 2001 census | Black |
| 285932018 | 9S41 | 9S41.00 | Black British | Black |
| 4919061000006110 | ^ESCTCA491906 |  | Caribbean country | Black |
| 1565711000006110 | EMISNQSO9 |  | South African | Black |
| 250243013 | 134K | 134K.00 | Race: West indian | Black |
| 459730016 | ESCTBL5 |  | Black - ethnic group | Black |
| 285949013 | 9S47 | 9S47.00 | Black - other Asian | Black |
| 453110019 | 9S42-3 | 9S42.13 | Black Guyana | Black |
| 1968571000006110 | 9t2G | 9t2G.00 | Carib/Black: any other Black/Caribbean grp - Scotland 2011 | Black |
| 405065012 | 9S43 | 9S43.00 | Black N African/Arab/Iranian | Black |
| 285971015 | 9SA5 | 9SA5.00 | Other African countries (NMO) | Black |
| 411578016 | 9SA3-1 | 9SA3.11 | Caribbean Island (NMO) | Black |
| 196601000006113 | 134H | 134H.00 | RACE: Afro-caribbean | Black |
| 154401000000118 | 9iB | 9iB..00 | Caribbean - ethnic category 2001 census | Black |
| 1564491000006110 | EMISNQCE3 |  | Central African | Black |
| 157311000000119 | 9iD2 | 9iD2.00 | Black British - ethnic category 2001 census | Black |
| 285931013 | 9S4 | 9S4..00 | Black, other, non-mixed origin | Black |
| 1968561000006110 | 9t2F | 9t2F.00 | Carib/Black: Black/Black Scot/Black Brit- Scotland 2011 cens | Black |
| 5516681000006110 | ^ESCTBL551668 |  | Black Caribbean/West India/Guyana | Black |
| 411573013 | 9S43-1 | 9S43.11 | Black North African | Black |
| 285930014 | 9S42-1 | 9S42.11 | Black Caribbean | Black |
| 411577014 | 9S45-2 | 9S45.12 | Black Indo-Caribbean | Black |
| 158371000000111 | 9iD3 | 9iD3.00 | Mixed Black - ethnic category 2001 census | Black |
| 250231012 | 134A | 134A.00 | West Indian origin | Black |
| 1968181000006110 | 9t0D | 9t0D.00 | Black/African/Carib/Black Brit: African- Eng+Wales 2011 cens | Black |
| 514651000006112 | 9S45 | 9S45.00 | Black E Afric Asia/Indo-Caribb | Black |
| 285948017 | 9S46 | 9S46.00 | Black Indian sub-continent | Black |
| 1968361000006110 | 9t1C | 9t1C.00 | Black/Afri/Carib/Black Brit: Caribbean- NI eth cat 2011 cens | Black |
| 937731000006115 | 9iD4 | 9iD4.00 | Other Black or Black unspecified ethnic category 2001 census | Black |
| 141381000000117 | 9iA | 9iA..00 | Other Asian background - ethnic category 2001 census | Asian |
| 1968171000006110 | 9t0C | 9t0C.00 | Asian/Asian Brit: other Asian- Eng+Wales eth cat 2011 census | Asian |
| 196671000006119 | 134F | 134F.00 | RACE: Korean | Asian |
| 141401000000117 | 9iE | 9iE..00 | Chinese - ethnic category 2001 census | Asian |
| 285956019 | 9S8 | 9S8..00 | Bangladeshi | Asian |
| 196631000006117 | 134I | 134I.00 | RACE: Bangladeshi | Asian |
| 1564731000006110 | EMISNQFI10 |  | Filipino | Asian |
| 141361000000114 | 9i8 | 9i8..00 | Pakistani or British Pakistani - ethnic category 2001 census | Asian |
| 6296681000006110 | ^ESCTSI629668 |  | Sikh, follower of religion | Asian |
| 1968141000006110 | 9t09 | 9t09.00 | Asian/Asian British:Pakistani- Eng+Wales eth cat 2011 census | Asian |
| 1968341000006110 | 9t1A | 9t1A.00 | Asian/Asian British: other Asian - NI ethnic cat 2011 census | Asian |
| 141511000000116 | 9iA9 | 9iA9.00 | Mixed Asian - ethnic category 2001 census | Asian |
| 141521000000110 | 9iA1 | 9iA1.00 | Punjabi - ethnic category 2001 census | Asian |
| 1968161000006110 | 9t0B | 9t0B.00 | Asian/Asian Brit: Chinese - Eng+Wales ethnic cat 2011 census | Asian |
| 157351000000115 | 9iF5 | 9iF5.00 | Hindu - ethnic category 2001 census | Asian |
| 141551000000117 | 9iA5 | 9iA5.00 | Tamil - ethnic category 2001 census | Asian |
| 1565731000006110 | EMISNQSR1 |  | Sri Lankan | Asian |
| 412016016 | 2263-1 | 2263.11 | O/E - Asian origin | Asian |
| 141641000000116 | 9iF2 | 9iF2.00 | Filipino - ethnic category 2001 census | Asian |
| 1564521000006110 | EMISNQCH30 |  | Chinese | Asian |
| 250230013 | 1349 | 1349 | Far Eastern origin | Asian |
| 141561000000119 | 9iA8 | 9iA8.00 | British Asian - ethnic category 2001 census | Asian |
| 158361000000116 | 9i64 | 9i64.00 | Asian and Chinese - ethnic category 2001 census | Asian |
| 1968331000006110 | 9t19 | 9t19.00 | Asian/Asian British: Chinese - NI ethnic cat 2011 census | Asian |
| 1725921000006110 | EMISNQCH53 |  | Chinese Evangelical Christian, follower of religion | Asian |
| 8119551000006110 | ^ESCTCH811955 |  | Chinese Evangelical Christian, follower of religion | Asian |
| 285955015 | 9S7 | 9S7..00 | Pakistani | Asian |
| 196651000006112 | 134D | 134D.00 | RACE: Chinese | Asian |
| 141651000000118 | 9iF3 | 9iF3.00 | Malaysian - ethnic category 2001 census | Asian |
| 1572831000000110 | 9T9 | 9T9..00 | Nepali | Asian |
| 56590016 | 9S9 | 9S9..00 | Chinese | Asian |
| 5580031000006110 | ^ESCTON558003 |  | On examination - Asian origin | Asian |
| 136081000000111 | 9iA4 | 9iA4.00 | Sri Lankan - ethnic category 2001 census | Asian |
| 141541000000115 | 9iA3 | 9iA3.00 | East African Asian - ethnic category 2001 census | Asian |
| 250228011 | 1347 | 1347 | Indian origin | Asian |
| 411583012 | 9SA6-1 | 9SA6.11 | East African Asian (NMO) | Asian |
| 1565011000006110 | EMISNQJA5 |  | Japanese | Asian |
| 285977016 | 9SA8 | 9SA8.00 | Other Asian (NMO) | Asian |
| 157301000000116 | 9iA6 | 9iA6.00 | Sinhalese - ethnic category 2001 census | Asian |
| 1968321000006110 | 9t18 | 9t18.00 | Asian/Asian British: Bangladeshi - NI ethnic cat 2011 census | Asian |
| 196721000006111 | 134M | 134M.00 | RACE: Pakistani | Asian |
| 4917181000006110 | ^ESCTEA491718 |  | East African country | Asian |
| 1968301000006110 | 9t16 | 9t16.00 | Asian or Asian British: Indian - NI ethnic cat 2011 census | Asian |
| 286018012 | 9T1B | 9T1B.00 | South East Asian | Asian |
| 285976013 | 9SA7 | 9SA7.00 | Indian sub-continent (NMO) | Asian |
| 490271012 | 135B | 135B.00 | Sikh | Asian |
| 459870015 | 13e | 13e..00 | Country of birth (Asian) | Asian |
| 4917591000006110 | ^ESCTAS491759 |  | Asian country | Asian |
| 405069018 | 9SA6 | 9SA6.00 | E Afric Asian/Indo-Carib (NMO) | Asian |
| 142891000000115 | 9iF8 | 9iF8.00 | Sikh - ethnic category 2001 census | Asian |
| 1968511000006110 | 9t2A | 9t2A.00 | Asian: Chinese - Scotland ethnic category 2011 census | Asian |
| 141631000000113 | 9iF1 | 9iF1.00 | Japanese - ethnic category 2001 census | Asian |
| 402434017 | 2263 | 2263 | O/E - Mongoloid origin | Asian |
| 1968521000006110 | 9t2B | 9t2B.00 | Asian: other Asian group - Scotland ethnic cat 2011 census | Asian |
| 1968481000006110 | 9t27 | 9t27.00 | Asian: Pakistani/Pakistani Scot/Pakistani Brit- Scot 2011 | Asian |
| 141531000000112 | 9iA2 | 9iA2.00 | Kashmiri - ethnic category 2001 census | Asian |
| 157271000000119 | 9i7 | 9i7..00 | Indian or British Indian - ethnic category 2001 census | Asian |
| 1968151000006110 | 9t0A | 9t0A.00 | Asian/Asian Brit: Bangladeshi- Eng+Wales eth cat 2011 census | Asian |
| 1968131000006110 | 9t08 | 9t08.00 | Asian/Asian Brit: Indian - Eng+Wales ethnic cat 2011 census | Asian |
| 4918001000006110 | ^ESCTSR491800 |  | Sri Lanka | Asian |
| 250224013 | 1343 | 1343 | Asian origin | Asian |
| 1968311000006110 | 9t17 | 9t17.00 | Asian/Asian British: Pakistani - NI ethnic cat 2011 census | Asian |
| 1565211000006110 | EMISNQMA9 |  | Malaysian | Asian |
| 285954016 | 9S6 | 9S6..00 | Indian | Asian |
| 456650013 | 9SC | 9SC..00 | Vietnamese | Asian |
| 411584018 | 9SA6-2 | 9SA6.12 | Indo-Caribbean (NMO) | Asian |
| 196661000006114 | 134E | 134E.00 | RACE: Japanese | Asian |
| 550541000006110 | 9T1C | 9T1C.00 | Chinese | Asian |
| 459784018 | 9SH | 9SH..00 | Other Asian ethnic group | Asian |
| 937651000006117 | 9iAA | 9iAA.00 | Other Asian or Asian unspecified ethnic category 2001 census | Asian |
| 1968491000006110 | 9t28 | 9t28.00 | Asian: Indian, Indian Scot/Indian Brit- Scotland 2011 census | Asian |
| 1565951000006110 | EMISNQVI7 |  | Vietnamese | Asian |
| 141621000000111 | 9iF0 | 9iF0.00 | Vietnamese - ethnic category 2001 census | Asian |
| 286020010 | 9T1E | 9T1E.00 | Other Asian | Asian |
| 438450019 | 13b1 | 13b1.00 | Cantonese Chinese dialect | Asian |
| 937541000006115 | 9i9 | 9i9..00 | Bangladeshi or British Bangladeshi - ethn categ 2001 census | Asian |
| 4917701000006110 | ^ESCTEA491770 |  | East Asian country | Asian |
| 196701000006118 | 134G | 134G.00 | RACE: Oriental | Asian |
| 937511000006119 | 9i65 | 9i65.00 | Other Mixed or Mixed unspecified ethnic category 2001 census | Mixed |
| 4740381000006110 | ^ESCTOT474038 |  | Other ethnic, mixed white origin | Mixed |
| 1968271000006110 | 9t13 | 9t13.00 | Mixed: White and Black African - NI ethnic cat 2011 census | Mixed |
| 157291000000115 | 9i62 | 9i62.00 | Black and White - ethnic category 2001 census | Mixed |
| 4740401000006110 | ^ESCTOT474040 |  | Other ethnic, other mixed origin | Mixed |
| 1968281000006110 | 9t14 | 9t14.00 | Mixed: White and Asian - NI ethnic category 2011 census | Mixed |
| 196611000006111 | 134L | 134L.00 | RACE: Afro-caucasian | Mixed |
| 141341000000113 | 9i5 | 9i5..00 | White and Asian - ethnic category 2001 census | Mixed |
| 1968091000006110 | 9t04 | 9t04.00 | Mixed: White+Black Caribbean - Eng+Wales eth cat 2011 census | Mixed |
| 158341000000117 | 9i0 | 9i0..00 | British or mixed British - ethnic category 2001 census | Mixed |
| 4740341000006110 | ^ESCTOT474034 |  | Other ethnic, Black/White origin | Mixed |
| 4740361000006110 | ^ESCTOT474036 |  | Other ethnic, Asian/White origin | Mixed |
| 285952017 | 9S51 | 9S51.00 | Other Black - Black/White orig | Mixed |
| 1968261000006110 | 9t12 | 9t12.00 | Mixed: White and Black Caribbean - NI ethnic cat 2011 census | Mixed |
| 1968111000006110 | 9t06 | 9t06.00 | Mixed: White+Asian - Eng+Wales ethnic category 2011 census | Mixed |
| 1968121000006110 | 9t07 | 9t07.00 | Mixed: other Mixed/multiple backgrd - Eng+Wales 2011 census | Mixed |
| 459729014 | ESCTMI5 |  | Mixed ethnic census group | Mixed |
| 285991010 | 9SB2 | 9SB2.00 | Other ethnic, Asian/White orig | Mixed |
| 285992015 | 9SB3 | 9SB3.00 | Other ethnic, mixed white orig | Mixed |
| 460154012 | 9SB6 | 9SB6.00 | Black African and White | Mixed |
| 460153018 | 9SB5 | 9SB5.00 | Black Caribbean and White | Mixed |
| 141331000000116 | 9i4 | 9i4..00 | White and Black African - ethnic category 2001 census | Mixed |
| 196681000006116 | 134J | 134J.00 | RACE: Mixed | Mixed |
| 1968101000006110 | 9t05 | 9t05.00 | Mixed: White+Black African - Eng+Wales eth cat 2011 census | Mixed |
| 141321000000118 | 9i3 | 9i3..00 | White and Black Caribbean - ethnic category 2001 census | Mixed |
| 285989019 | 9SB | 9SB..00 | Other ethnic, mixed origin | Mixed |
| 285993013 | 9SB4 | 9SB4.00 | Other ethnic, other mixed orig | Mixed |
| 142791000000111 | 9i2S | 9i2S.00 | Other mixed White - ethnic category 2001 census | Mixed |
| 1968471000006110 | 9t26 | 9t26.00 | Mixed/multiple ethnic grps: any- Scot ethnic cat 2011 census | Mixed |
| 285990011 | 9SB1 | 9SB1.00 | Other ethnic, Black/White orig | Mixed |
| 141471000000113 | 9i60 | 9i60.00 | Black and Asian - ethnic category 2001 census | Mixed |
| 141481000000110 | 9i61 | 9i61.00 | Black and Chinese - ethnic category 2001 census | Mixed |
| 141351000000111 | 9i6 | 9i6..00 | Other Mixed background - ethnic category 2001 census | Mixed |
| 141491000000112 | 9i63 | 9i63.00 | Chinese and White - ethnic category 2001 census | Mixed |
| 1968291000006110 | 9t15 | 9t15.00 | Mixed: other Mixed/multiple ethnic backgrd - NI 2011 census | Mixed |
| 286017019 | 9T1A | 9T1A.00 | Other Pacific ethnic group | Other |
| 411581014 | 9SA4-1 | 9SA4.11 | North African Arab (NMO) | Other |
| 405071018 | 9SAB | 9SAB.00 | Turkish/Turkish Cypriot (NMO) | Other |
| 142861000000114 | 9iFG | 9iFG.00 | Latin American - ethnic category 2001 census | Other |
| 142851000000111 | 9iFF | 9iFF.00 | Moroccan - ethnic category 2001 census | Other |
| 285958018 | 9SA | 9SA..00 | Other ethnic non-mixed (NMO) | Other |
| 4740241000006110 | ^ESCTBR474024 |  | British ethnic minority specified (NMO) | Other |
| 4740261000006110 | ^ESCTBR474026 |  | British ethnic minority unspecified (NMO) | Other |
| 1598531000000110 | 918t | 918t.00 | Carer from Black and minority ethnic group | Other |
| 142831000000116 | 9iFC | 9iFC.00 | Israeli - ethnic category 2001 census | Other |
| 411597016 | 9SAB-2 | 9SAB.12 | Turkish Cypriot (NMO) | Other |
| 405068014 | 9SA4 | 9SA4.00 | N African Arab/Iranian (NMO) | Other |
| 250227018 | 1346 | 1346 | Australian origin | Other |
| 142901000000119 | 9iFK | 9iFK.00 | Any other group - ethnic category 2001 census | Other |
| 286014014 | 9T17 | 9T17.00 | Niuean | Other |
| 286021014 | 9T1Y | 9T1Y.00 | Other New Zealand ethnic group | Other |
| 285988010 | 9SAD | 9SAD.00 | Other ethnic NEC (NMO) | Other |
| 286015010 | 9T18 | 9T18.00 | Tokelauan | Other |
| 285960016 | 9SA2 | 9SA2.00 | Brit. ethnic minor. unsp (NMO) | Other |
| 1968391000006110 | 9t1F | 9t1F.00 | Other ethnic group: any other grp- NI ethnic cat 2011 census | Other |
| 411596013 | 9SAB-1 | 9SAB.11 | Turkish (NMO) | Other |
| 1968581000006110 | 9t2H | 9t2H.00 | Other ethnic grp: Arab/Arab Scot/Arab British- Scotland 2011 | Other |
| 250226010 | 1345 | 1345 | South American origin | Other |
| 142811000000112 | 9iFA | 9iFA.00 | North African - ethnic category 2001 census | Other |
| 142841000000113 | 9iFE | 9iFE.00 | Kurdish - ethnic category 2001 census | Other |
| 459785017 | 9SJ | 9SJ..00 | Other ethnic group | Other |
| 937941000006111 | 9iFJ | 9iFJ.00 | Mauritian/Seychellois/Maldivian/St Helena eth cat 2001census | Other |
| 507015012 | 9T14 | 9T14.00 | Samoan | Other |
| 2615361000000110 | 9TC4 |  | Slovak Roma | Other |
| 250229015 | 1348 | 1348 | Middle Eastern origin | Other |
| 937871000006114 | 9iFB | 9iFB.00 | Mid East (excl Israeli, Iranian & Arab) - eth cat 2001 cens | Other |
| 1565321000006110 | EMISNQMO16 |  | Moroccan | Other |
| 2645811000000110 | 9TC |  | Roma ethnic group | Other |
| 138251000000111 | 9iF9 | 9iF9.00 | Arab - ethnic category 2001 census | Other |
| 1968221000006110 | 9t0H | 9t0H.00 | Other ethnic: any other grp - Eng+Wales eth cat 2011 census | Other |
| 1968381000006110 | 9t1E | 9t1E.00 | Other ethnic group: Arab - NI ethnic category 2011 census | Other |
| 1968211000006110 | 9t0G | 9t0G.00 | Other ethnic group: Arab - Eng+Wales ethnic cat 2011 census | Other |
| 1565391000006110 | EMISNQNE21 |  | New Zealander | Other |
| 286013015 | 9T15 | 9T15.00 | Cook Island Maori | Other |
| 141411000000115 | 9iF | 9iF..00 | Other - ethnic category 2001 census | Other |
| 285959014 | 9SA1 | 9SA1.00 | Brit. ethnic minor. spec.(NMO) | Other |
| 371005013 | 134P-1 | 134P.11 | Race: Other | Other |
| 142721000000113 | 9i2A | 9i2A.00 | Turkish Cypriot - ethnic category 2001 census | Other |
| 1565961000006110 | EMISNQYE1 |  | Yemeni | Other |
| 1565591000006110 | EMISNQSA9 |  | Samoan | Other |
| 250225014 | 1344 | 1344 | North American origin | Other |
| 501416013 | 9T19 | 9T19.00 | Fijian | Other |
| 523591000000116 | 9T3 | 9T3..00 | Yemeni | Other |
| 411582019 | 9SA4-2 | 9SA4.12 | Iranian (NMO) | Other |
| 138271000000119 | 9iFH | 9iFH.00 | South and Central American - ethnic category 2001 census | Other |
| 286022019 | 9T1Z | 9T1Z.00 | New Zealand ethnic group NOS | Other |
| 138261000000114 | 9iFD | 9iFD.00 | Iranian - ethnic category 2001 census | Other |
| 286012013 | 9T13 | 9T13.00 | New Zealand Maori | Other |
| 1564721000006110 | EMISNQFI9 |  | Fijian | Other |
| 286006015 | 9T1 | 9T1..00 | New Zealand ethnic groups | Other |
| 253634013 | 2264 | 2264 | O/E - Australoid | Other |
| 196621000006115 | 134C | 134C.00 | RACE: Arab | Other |
| 286008019 | 9T11-1 | 9T11.11 | Pakeha | Other |
| 1968591000006110 | 9t2J | 9t2J.00 | Other ethnic grp: any other ethnic grp- Scotland 2011 census | Other |
| 8113231000006110 | ^ESCTSL811323 |  | Slovak language interpreter needed | ~white |
| 642141000000118 | 13u0 | 13u0.00 | Main spoken language Bulgarian | ~white |
| 4918451000006110 | ^ESCTSL491845 |  | Slovakia | ~white |
| 8119471000006110 | ^ESCTBU811947 |  | Bulgarian Orthodox, follower of religion | ~white |
| 310761000000119 | 9NUA | 9NUA.00 | Interpreter needed - Czech | ~white |
| 460086017 | 13l3 | 13l3.00 | Main spoken language Czech | ~white |
| 8063501000006110 | ^ESCTCZ806350 |  | Czech language interpreter needed | ~white |
| 1861951000006110 | EMISNQPR251 |  | Preferred written language: Bulgarian | ~white |
| 1656481000006110 | EMISNQMA18 |  | Main spoken language Bulgarian | ~white |
| 1778711000006110 | EMISNQPR112 |  | Preferred communication Language: Portuguese | ~white |
| 4918401000006110 | ^ESCTCZ491840 |  | Czech republic | ~white |
| 1862951000006110 | EMISNQPR351 |  | Preferred written language: Slovak | ~white |
| 937311000006113 | 9i2H | 9i2H.00 | Commonwealth (Russian) Indep States - ethn categ 2001 census | ~white |
| 993241000006119 | EMISNQLA9 |  | Language of interpreter - Czech | ~white |
| 1696131000006110 | EMISNQMA26 |  | Main spoken language Slovak | ~white |
| 993361000006113 | EMISNQLA21 |  | Language of interpreter - Portuguese | ~white |
| 8064141000006110 | ^ESCTPO806414 |  | Portuguese language interpreter needed | ~white |
| 1854451000006110 | EMISNQPR194 |  | Preferred written language: Portuguese | ~white |
| 314081000000115 | 13ng | 13ng.00 | Reads Bulgarian | ~white |
| 311811000000116 | 9NUb | 9NUb.00 | Interpreter needed - Portuguese | ~white |
| 662801000000116 | 13uL | 13uL.00 | Main spoken language Slovak | ~white |
| 459927016 | 13dA | 13dA.00 | Born in Czech Republic | ~white |
| 460098010 | 13lD | 13lD.00 | Main spoken language Portuguese | ~white |
| 216038018 | 13n1 | 13n1.00 | Reads Portuguese | ~white |
| 216044019 | 13nA | 13nA.00 | Reads Czech | ~white |
| 1726231000006110 | EMISNQBU8 |  | Bulgarian Orthodox, follower of religion | ~white |
| 1862081000006110 | EMISNQPR264 |  | Preferred written language: Czech | ~white |
| 713371000000117 | 13z1 | 13z1.00 | Bulgarian Orthodox | ~white |
| 4917381000006110 | ^ESCTWE491738 |  | West African country | ~Black |
| 713191000000116 | 135l | 135l.00 | African religion, follower of religion | ~Black |
| 4917051000006110 | ^ESCTCE491705 |  | Central African country | ~Black |
| 4917291000006110 | ^ESCTSO491729 |  | South west African country | ~Black |
| 4917321000006110 | ^ESCTSO491732 |  | South African country | ~Black |
| 4917251000006110 | ^ESCTSO491725 |  | South east African country | ~Black |
| 4917081000006110 | ^ESCTCE491708 |  | Central African Republic | ~Black |
| 6057371000006110 | ^ESCTCO605737 |  | Country of birth - African | ~Black |
| 1778871000006110 | EMISNQPR128 |  | Preferred communication Language: Vietnamese | ~Asian |
| 216045018 | 13nE | 13nE.00 | Reads Chinese | ~Asian |
| 992091000006110 | EMISNQJA2 |  | Japanese buddhist religion | ~Asian |
| 8063811000006110 | ^ESCTJA806381 |  | Japanese language interpreter needed | ~Asian |
| 312441000000114 | 9NUt | 9NUt.00 | Interpreter needed - Vietnamese | ~Asian |
| 5847801000006110 | ^ESCTJA584780 |  | Japanese buddhism | ~Asian |
| 4917911000006110 | ^ESCTSO491791 |  | South Asian country | ~Asian |
| 1854141000006110 | EMISNQPR163 |  | Preferred written language: Cantonese and Vietnamese | ~Asian |
| 4918031000006110 | ^ESCTSO491803 |  | South west Asian country | ~Asian |
| 6049901000006110 | ^ESCTTH604990 |  | Third Japanese encephalitis vaccination | ~Asian |
| 1862751000006110 | EMISNQPR331 |  | Preferred written language: Nepali | ~Asian |
| 673551000000118 | 13w1 | 13w1.00 | Main spoken language Nepali | ~Asian |
| 437977019 | 13b0 | 13b0.00 | Vietnamese language | ~Asian |
| 2159254019 | 13lW | 13lW.00 | Main spoken language Japanese | ~Asian |
| 1854551000006110 | EMISNQPR204 |  | Preferred written language: Tagalog (Filipino) | ~Asian |
| 992011000006117 | EMISNQCH4 |  | Chinese buddhist religion | ~Asian |
| 1854611000006110 | EMISNQPR210 |  | Preferred written language: Vietnamese | ~Asian |
| 8064501000006110 | ^ESCTTA806450 |  | Tamil language interpreter needed | ~Asian |
| 1778821000006110 | EMISNQPR123 |  | Preferred communication Language: Tamil | ~Asian |
| 687071000000116 | 9NnK | 9NnK.00 | Nepali language interpreter needed | ~Asian |
| 8355881000006110 | ^ESCTMA835588 |  | Main spoken language Chinese | ~Asian |
| 460105017 | 13lK | 13lK.00 | Main spoken language Tamil | ~Asian |
| 4917601000006110 | ^ESCTNO491760 |  | North and central Asian country | ~Asian |
| 311331000000119 | 9NUP | 9NUP.00 | Interpreter needed - Japanese | ~Asian |
| 312261000000111 | 9NUn | 9NUn.00 | Interpreter needed - Tamil | ~Asian |
| 1854561000006110 | EMISNQPR205 |  | Preferred written language: Tamil | ~Asian |
| 1778591000006110 | EMISNQPR100 |  | Preferred communication Language: Japanese | ~Asian |
| 1862021000006110 | EMISNQPR258 |  | Preferred written language: Chinese | ~Asian |
| 6057331000006110 | ^ESCTCO605733 |  | Country of birth - Asian | ~Asian |
| 992001000006115 | EMISNQCE2 |  | Central Asian buddhist religion | ~Asian |
| 1854341000006110 | EMISNQPR183 |  | Preferred written language: Japanese | ~Asian |
| 1778391000006110 | EMISNQPR80 |  | Preferred communication Language: Cantonese and Vietnamese | ~Asian |
| 460044011 | 13ef | 13ef.00 | Born in Sri Lanka | ~Asian |
| 1778811000006110 | EMISNQPR122 |  | Preferred communication Language: Tagalog (Filipino) | ~Asian |
| 1706071000006110 | EMISNQMA35 |  | Main spoken language Chinese | ~Asian |
| 2159261015 | 13lb | 13lb.00 | Main spoken language Vietnamese | ~Asian |
| 8064641000006110 | ^ESCTVI806464 |  | Vietnamese language interpreter needed | ~Asian |
| 1862181000006110 | EMISNQPR274 |  | Preferred written language: Filipino | ~Asian |
| 4917771000006110 | ^ESCTSO491777 |  | South east Asian country | ~Asian |
| 5847841000006110 | ^ESCTCE584784 |  | Central Asian buddhism | ~Asian |
| 1118721000000110 | 13w4 | 13w4.00 | Main spoken language Filipino | ~Asian |
| 4917011000006110 | ^ESCTNO491701 |  | North east African country | ~Other |
| 1862171000006110 | EMISNQPR273 |  | Preferred written language: Fijian | ~Other |
| 664991000000112 | 9NUz | 9NUz.00 | Bulgarian language interpreter needed | ~Other |
| 2615691000000110 | 9TC0 |  | Bulgarian Roma | ~Other |
| 686231000000112 | 9NnD | 9NnD.00 | Samoan language interpreter needed | ~Other |
| 4916951000006110 | ^ESCTNO491695 |  | North African country | ~Other |
| 4918281000006110 | ^ESCTNE491828 |  | New Zealand | ~Other |
| 1862881000006110 | EMISNQPR344 |  | Preferred written language: Samoan | ~Other |
| 6057391000006110 | ^ESCTCO605739 |  | Country of birth - Australasian | ~Other |
| 671491000000112 | 13uS | 13uS.00 | Main spoken language Fijian | ~Other |
| 673911000000114 | 13w7 | 13w7.00 | Main spoken language Samoan | ~Other |
| 459873018 | 13h | 13h..00 | Country of birth (Australasian) | ~Other |
| 2361401000000110 | 13kE | 13kE.00 | Born in Cook Islands | ~Other |
| 460005015 | 13h1 | 13h1.00 | Born in New Zealand | ~Other |
| 460037019 | 13dc | 13dc.00 | Born in Slovakia | ~Other |
| 681931000000111 | 9NmW | 9NmW.00 | Fijian language interpreter needed | ~Other |
| 142881000000117 | 9iF4 | 9iF4.00 | Buddhist - ethnic category 2001 census | ~Other |
