# Supplementary material for: Ethnic inequalities in routes to diagnosis of cancer: a population-based UK cohort study
Source: Br J Cancer. 2022 Jun 6;127(5):863–71. doi: 10.1038/s41416-022-01847-x (PMC9427836; doi:10.1038/s41416-022-01847-x)
Supplement: Supplementary file 3 — Supplementary File 2 - revised [file 41416_2022_1847_MOESM3_ESM.docx]

**Supplementary File 2: Flowchart showing the process of identifying patients’ ethnicity**

Unclear latest or frequent records (n=2,435)

Has any ethnicity record (n=471,128)

**Potential participants**

(N = 590,235)

Has multiple ethnicity records (n=373,594)

Has multiple ethnicity records (n=104,798)

Duplicates removed (n=268,796)

Duplicates removed (n=1,220)

**Include**

Replace unclear- latest/most frequent with HES data (n=1,199)

**Exclude**

Missing in CPRD

& HES (n=16)

**Include**

Has single usable ethnicity record (n=97,534)

**Include**

Most frequent (n=84,197)

Latest (n=18,166)

**Include**

Replace missing/unusable records with HES data

(n=98,536)

Duplicate removed (n=20,571)

Missing ethnicity (n=93,505)

Unusable ethnicity (n=25,602)

Total with ethnicity records in CPRD

(n=199,897)

**Exclude**

Other cancers (n=53,072)

Final eligible patients with ethnicity and 10 cancers

(N=244,731)

Total with ethnicity records in CPRD and HES (n=297,803)

**Exclude**

Missing in CPRD & HES (n=1,829)
