# Supplementary material for: Ethnic inequalities in routes to diagnosis of cancer: a population-based UK cohort study
Source: Br J Cancer. 2022 Jun 6;127(5):863–71. doi: 10.1038/s41416-022-01847-x (PMC9427836; doi:10.1038/s41416-022-01847-x)
Supplement: Supplementary file 4 — Supplementary file 3 [file 41416_2022_1847_MOESM4_ESM.docx]

**Supplementary file 3: Ethnic differences in routes to diagnosis by patient characteristic**

**3a. Ethnic differences in Emergency Route**

| **Covariates** | **White*, %***  **N=28,963** | **Black, %**  **N=680** | **Asian, %**  **N=606** | **Mixed, %**  **N=13,070** | **Other, %**  **N=1,112** | **Total, n (%)** |
| --- | --- | --- | --- | --- | --- | --- |
| Gender  Female  *Male* | 18.6  19.7 | 13.3  15.7 | 10.3  17.4 | 15.9  16.9 | 27.9  28.2 | 44,431  (18.2) |
| *Age groups*  *40-49*  *50-59*  *60-69*  *≥70* | 9.76  11.5  13.2  25.7 | 6.53  11.0  10.9  22.4 | 9.09  9.46  9.94  21.0 | 8.23  9.51  11.5  22.0 | 12.9  17.8  23.8  34.8 |  |
| IMD  *Least Deprived 1*  *2*  *3*  *4*  *Most Deprived 5* | 15.5  17.2  19.0  21.7  25.2 | 7.14  15.3  14.5  14.9  15.5 | 9.96  10.8  12.0  16.4  16.6 | 12.7  15.1  16.1  18.5  21.4 | 24.8  28.9  28.3  28.1  31.9 |  |
| Morbidity Scores  *Least Morbidity Score 0*  *1*  *2*  *3*  *Most Morbidity Score 4* | 13.1  11.9  18.6  21.9  26.1 | 9.70  9.46  12.5  18.7  21.2 | 9.37  9.51  13.1  14.2  19.4 | 8.98  8.97  14.6  18.9  22.8 | 21.2  19.9  31.7  33.5  35.9 |  |
| Region  *North East*  *North West*  *Yorkshire*  *East Midlands*  *West Midlands*  *East of England*  *South West*  *South Central*  *London*  *South East* | 22.6  20.7  20.2  17.7  18.2  16.3  18.9  17.8  20.9  18.4 | 12.5  16.7  8.33  13.2  16.0  11.4  13.3  9.55  14.9  11.8 | 7.84  14.2  19.4  12.1  15.2  9.04  7.84  10.9  13.8  13.6 | 20.5  16.8  17.7  14.2  14.6  13.2  16.0  15.9  20.8  14.9 | 36.0  40.8  38.8  21.3  23.7  25.9  30.5  21.5  20.5  27.7 |  |
| Cancer Site  *Breast*  *Lung*  *Prostate*  *Colorectal*  *Oesophagus*  *stomach*  *Oral*  *Ovary*  *Myeloma*  *Cervix* | 4.34  37.1  8.60  25.0  20.7  30.8  7.02  30.6  35.6  14.1 | 2.62  36.2  7.22  23.2  24.6  33.0  11.5  40.3  26.2  18.4 | 1.99  33.2  6.53  18.2  21.5  36.7  2.67  26.4  31.3  7.69 | 3.37  34.5  7.39  21.6  18.4  29.8  5.96  29.1  33.9  10.9 | 8.76  43.7  12.6  33.6  24.6  37.9  9.30  40.3  36.4  25.7 |  |

**3b. Ethnic differences in Elective GP Referral Route**

| **Covariates** | **White*, %***  **N=34,680** | **Black, %**  **N=1,428** | **Asian, %**  **N=1,273** | **Mixed, %**  **N=18,428** | **Other, %**  **N=771** | **Total, n (%)** |
| --- | --- | --- | --- | --- | --- | --- |
| Gender  Female  *Male* | 17.1  28.7 | 22.3  36.4 | 21.6  36.5 | 16.4  29.8 | 17.0  22.0 | 56,580  (23.2) |
| *Age groups*  *40-49*  *50-59*  *60-69*  *≥70* | 21.4  21.2  23.8  23.3 | 31.7  31.2  31.9  30.3 | 27.1  25.6  27.8  31.6 | 20.1  21.5  23.2  23.9 | 27.7  19.6  18.5  18.9 |  |
| IMD  *Least Deprived 1*  *2*  *3*  *4*  *Most Deprived 5* | 23.8  23.5  23.3  22.1  21.3 | 35.7  29.9  28.1  31.9  31.3 | 29.5  28.5  28.9  26.9  28.9 | 24.6  23.5  23.1  21.9  22.2 | 19.4  21.8  18.2  18.6  18.8 |  |
| Morbidity Scores  *Least Morbidity Score 0*  *1*  *2*  *3*  *Most Morbidity Score 4* | 19.5  22.6  22.5  23.9  24.6 | 29.8  32.4  31.4  30.2  31.2 | 26.6  27.5  27.8  29.1  30.7 | 18.6  21.5  22.6  24.5  24.9 | 19.2  20.9  18.8  18.3  20.5 |  |
| Region  *North East*  *North West*  *Yorkshire*  *East Midlands*  *West Midlands*  *East of England*  *South West*  *South Central*  *London*  *South East* | 19.9  22.5  23.5  21.8  23.4  25.5  20.4  23.1  24.7  24.5 | 18.8  34.9  41.7  33.9  32.3  32.9  28.9  21.7  30.9  25.8 | 29.4  27.0  17.9  19.8  28.2  35.5  21.6  26.7  30.4  26.5 | 20.7  23.5  24.0  21.4  24.0  26.1  20.7  23.0  25.1  25.6 | 14.0  17.9  16.4  27.9  20.7  20.2  14.9  17.1  24.1  22.7 |  |
| Cancer Site  *Breast*  *Lung*  *Prostate*  *Colorectal*  *Oesophagus*  *stomach*  *Oral*  *Ovary*  *Myeloma*  *Cervix* | 10.1  21.6  38.0  22.5  18.7  22.1  26.9  23.3  32.9  27.9 | 16.0  22.2  42.3  28.8  21.7  21.1  35.4  27.3  38.0  48.9 | 12.9  31.5  48.9  29.8  29.9  23.4  34.6  27.4  36.9  42.3 | 9.31  22.2  38.3  23.4  18.5  21.6  27.9  22.4  35.0  25.9 | 14.2  17.4  30.4  18.7  15.7  17.3  25.6  24.5  23.9  28.6 |  |

**3c. Ethnic differences in Hospital Routes**

| **Covariates** | **White, %**  **N=15,539** | **Black, %**  **N=453** | **Asian, (%)**  **N=438** | **Mixed, %**  **N=8,174** | **Other, %**  **N=379** | **Total, n (%)** |
| --- | --- | --- | --- | --- | --- | --- |
| **Gender**  Female  *Male* | 7.96  12.6 | 8.36  10.8 | 7.30  12.7 | 7.84  12.7 | 8.11  11.1 | 24,983  (10.3) |
| ***Age groups***  *40-49*  *50-59*  *60-69*  *≥70* | 8.40  10.6  10.9  10.2 | 9.80  9.88  10.0  9.75 | 7.65  9.36  11.7  9.60 | 8.25  9.59  10.6  10.5 | 8.12  12.4  10.6  8.51 |  |
| **IMD**  *Least Deprived 1*  *2*  *3*  *4*  *Most Deprived 5* | 10.8  10.1  9.69  10.2  10.5 | 12.8  7.82  8.77  10.2  10.1 | 10.7  10.8  10.8  8.42  8.98 | 10.7  10.2  9.83  10.6  10.2 | 9.40  8.13  9.38  11.7  9.94 |  |
| **Morbidity Scores**  *Least Morbidity Score 0*  *1*  *2*  *3*  *Most Morbidity Score 4* | 9.30  9.69  10.3  10.9  10.7 | 9.38  9.11  9.83  9.50  11.2 | 8.52  8.48  9.13  11.0  11.0 | 8.31  9.24  10.2  10.9  11.1 | 10.6  8.32  10.3  8.98  9.60 |  |
| **Region**  *North East*  *North West*  *Yorkshire*  *East Midlands*  *West Midlands*  *East of England*  *South West*  *South Central*  *London*  *South East* | 10.4  10.5  8.19  8.99  10.0  10.5  10.5  11.8  9.53  9.94 | 25.0  8.85  12.5  13.2  10.7  12.7  11.1  14.0  9.16  12.9 | 13.7  8.66  5.97  8.79  9.84  10.2  8.50  12.2  10.2  7.53 | 9.90  10.0  8.52  10.4  9.81  9.26  10.9  12.7  9.54  9.90 | 6.00  9.32  2.24  9.84  10.4  10.1  9.00  10.1  11.9  8.78 |  |
| **Cancer Site**  *Breast*  *Lung*  *Prostate*  *Colorectal*  *Oesophagus*  *stomach*  *Oral*  *Ovary*  *Myeloma*  *Cervix* | 2.74  12.1  11.7  11.4  16.5  15.4  19.2  13.1  11.9  7.69 | 3.14  16.8  8.79  11.6  20.3  15.3  22.9  11.7  12.6  6.12 | 2.84  13.3  8.82  12.7  18.7  15.8  26.2  9.64  11.9  7.69 | 2.62  12.3  11.7  11.6  18.0  17.5  19.4  12.7  10.8  9.18 | 2.83  9.38  10.3  11.7  15.6  10.3  22.1  10.1  14.8  11.4 |  |

**3d. Ethnic differences in Screening Route**

| **Covariates** | **White*,* %**  **N=12,500** | **Black, %**  **N=235** | **Asian, %**  **N=487** | **Mixed, %**  **N=7,551** | **Other, %**  **N=197** | **Total, n (%)** |
| --- | --- | --- | --- | --- | --- | --- |
| **Gender**  Female  *Male* | 14.8  1.94 | 12.5  0.60 | 18.0  2.66 | 17.1  2.03 | 8.96  0.87 | 20,970  (8.61) |
| ***Age groups***  *40-49*  *50-59*  *60-69*  *≥70* | 5.22  17.3  15.4  2.41 | 2.84  9.79  7.85  1.71 | 3.90  18.6  18.2  3.39 | 5.93  19.7  17.7  2.67 | 5.17  12.1  10.2  0.65 |  |
| **IMD***  *Least Deprived 1*  *2*  *3*  *4*  *Most Deprived 5* | 9.66  8.95  8.41  7.39  5.93 | 4.59  7.48  6.58  4.98  4.33 | 12.3  12.7  11.8  9.31  9.36 | 10.8  10.2  9.96  8.65  7.09 | 6.88  4.51  5.47  3.14  4.01 |  |
| **Morbidity Scores**  *Least Morbidity Score 0*  *1*  *2*  *3*  *Most Morbidity Score 4* | 13.1  13.1  8.76  6.08  3.48 | 5.26  6.31  6.02  5.89  2.00 | 11.4  12.7  13.4  11.4  5.72 | 16.7  15.7  11.2  7.30  3.91 | 8.03  7.96  4.80  1.97  0.91 |  |
| **Region**  *North East*  *North West*  *Yorkshire*  *East Midlands*  *West Midlands*  *East of England*  *South West*  *South Central*  *London*  *South East* | 7.75  7.77  8.05  8.82  8.18  8.75  8.54  9.17  7.58  8.64 | 0  5.99  0  1.89  3.71  7.59  3.33  8.92  5.17  8.60 | 5.88  9.07  14.9  16.5  10.7  8.43  13.7  10.9  10.9  13.3 | 8.37  9.70  9.06  10.3  10.2  9.93  9.27  9.70  7.51  10.6 | 6.00  1.65  7.46  0  4.77  5.26  5.17  8.16  6.07  4.30 |  |
| **Cancer Site**  *Breast*  *Lung*  *Prostate*  *Colorectal*  *Oesophagus*  *stomach*  *Oral*  *Ovary*  *Myeloma*  *Cervix* | 29.3  -  -  7.69  -  -  -  -  -  18.8 | 20.5  -  -  5.47  -  -  -  -  -  12.2 | 28.3  -  -  10.7  -  -  -  -  -  19.2 | 31.7  -  -  8.35  -  -  -  -  -  22.1 | 22.1  -  -  3.47  -  -  -  -  -  14.3 |  |

**3e. Ethnic differences in TWW** **Route**

| **Covariates** | **White*,* *%***  **N=54,473** | **Black, %**  **N=1,683** | **Asian, %**  **N=1,494** | **Mixed, %**  **N=29,798** | **Other, %**  **N=1,167** | **Total, n (%)** |
| --- | --- | --- | --- | --- | --- | --- |
| **Gender**  Female  *Male* | 38.1  34.0 | 40.9  33.9 | 39.0  26.9 | 39.5  35.5 | 29.5  29.5 | 88,615 (36.4) |
| ***Age groups***  *40-49*  *50-59*  *60-69*  *≥70* | 49.6  35.1  33.7  35.8 | 46.2  35.7  35.1  34.3 | 47.3  32.6  28.6  31.5 | 52.6  35.8  33.8  38.0 | 36.9  30.5  29.9  28.1 |  |
| IMD*  *Least Deprived 1*  *2*  *3*  *4*  *Most Deprived 5* | 35.5  36.7  36.6  36.2  35.1 | 37.8  35.0  37.8  35.8  36.8 | 31.1  33.8  31.9  35.8  33.9 | 36.7  37.4  37.9  38.1  37.2 | 27.4  27.7  30.9  32.2  30.8 |  |
| **Morbidity Scores**  *Least Morbidity Score 0*  *1*  *2*  *3*  *Most Morbidity Score 4* | 40.1  38.4  36.6  34.7  32.9 | 41.5  39.9  37.6  33.4  32.9 | 37.7  36.4  33.2  31.6  30.8 | 42.7  40.0  38.4  35.8  34.9 | 32.6  32.3  27.9  28.9  24.5 |  |
| **Region**  *North East*  *North West*  *Yorkshire*  *East Midlands*  *West Midlands*  *East of England*  *South West*  *South Central*  *London*  *South East* | 37.6  35.9  37.4  40.3  37.2  34.9  38.6  34.4  33.4  34.2 | 37.5  32.6  37.5  35.9  35.3  34.2  40.0  42.7  36.9  38.7 | 37.3  37.3  38.8  41.8  33.5  30.7  43.1  34.2  31.0  33.7 | 39.0  37.5  38.6  40.3  38.1  37.1  43.1  34.6  32.9  34.2 | 30.0  23.8  27.6  36.1  30.8  25.9  31.4  35.2  31.2  26.7 |  |
| **Cancer Site**  *Breast*  *Lung*  *Prostate*  *Colorectal*  *Oesophagus*  *stomach*  *Oral*  *Ovary*  *Myeloma*  *Cervix* | 49.5  26.6  37.9  30.5  41.4  29.1  44.1  29.5  16.9  28.4 | 55.5  23.0  38.9  28.5  27.5  26.3  22.9  19.5  21.4  14.3 | 49.6  19.2  31.7  25.7  26.2  20.3  30.8  30.9  17.5  21.2 | 49.2  28.1  38.8  32.3  42.6  28.4  44.2  32.1  18.5  29.9 | 42.1  22.5  35.3  25.2  40.3  24.3  39.5  15.1  13.6  17.1 |  |

| **Covariates** | **White*,* *%***  **N=4,908** | **Black, %**  **N=119** | **Asian, %**  **N=170** | **Mixed, %**  **N=29,798** | **Other, %**  **N=1,167** | **Total, n (%)** |
| --- | --- | --- | --- | --- | --- | --- |
| **Gender**  Female  *Male* | 3.42  3.08 | 2.69  2.53 | 3.80  3.81 | 3.30  3.11 | 8.46  8.36 | 8,078  (3.25) |
| ***Age groups***  *40-49*  *50-59*  *60-69*  *≥70* | 5.63  4.34  3.11  2.70 | 2.98  2.42  4.17  1.65 | 4.91  4.34  3.82  2.94 | 4.96  3.90  3.23  2.78 | 9.23  7.71  7.03  9.12 |  |
| **IMD**  *Least Deprived 1*  *2*  *3*  *4*  *Most Deprived 5* | 4.67  3.50  2.93  2.36  2.01 | 2.04  4.42  4.26  2.19  1.99 | 6.50  3.38  4.64  3.07  2.20 | 4.63  3.66  3.03  2.21  1.98 | 12.1  9.01  7.81  6.29  4.65 |  |
| **Morbidity Scores**  *Least Morbidity Score 0*  *1*  *2*  *3*  *Most Morbidity Score 4* | 4.84  4.44  3.14  2.53  2.24 | 4.44  2.80  2.66  2.28  1.47 | 6.47  5.40  3.46  2.82  2.38 | 4.76  4.57  3.09  2.64  2.45 | 8.41  10.5  6.53  8.36  8.70 |  |
| **Region**  *North East*  *North West*  *Yorkshire*  *East Midlands*  *West Midlands*  *East of England*  *South West*  *South Central*  *London*  *South East* | 1.75  2.66  2.65  2.31  3.04  4.11  3.09  3.77  3.85  4.30 | 6.25  1.04  0  1.89  1.93  1.27  3.33  3.18  2.93  2.15 | 5.88  3.71  2.99  1.10  2.64  6.02  5.23  5.02  3.79  5.38 | 1.51  2.43  2.11  3.35  3.29  4.40  3.04  4.06  4.11  4.82 | 8.00  6.62  7.46  4.92  9.70  12.6  9.00  7.83  6.28  9.81 |  |
| **Cancer Site**  *Breast*  *Lung*  *Prostate*  *Colorectal*  *Oesophagus*  *stomach*  *Oral*  *Ovary*  *Myeloma*  *Cervix* | 3.98  2.60  3.75  2.85  2.75  2.71  2.76  3.61  2.71  3.03 | 2.20  1.82  2.76  2.32  5.80  4.31  7.29  1.30  1.85  0 | 4.26  2.83  3.90  2.87  3.74  3.80  5.77  5.58  2.50  1.92 | 3.79  2.48  3.88  2.82  2.42  2.68  2.59  3.74  1.80  2.04 | 10.1  7.04  11.4  7.32  4.27  10.3  3.49  10.1  11.4  2.86 |  |

**3f. Ethnic differences in Other Route**
