# Supplementary material for: Ethnic inequalities in routes to diagnosis of cancer: a population-based UK cohort study
Source: Br J Cancer. 2022 Jun 6;127(5):863–71. doi: 10.1038/s41416-022-01847-x (PMC9427836; doi:10.1038/s41416-022-01847-x)
Supplement: Supplementary file 5 — Supplementary file 4 [file 41416_2022_1847_MOESM5_ESM.docx]

**Supplementary file 4: Cancer-specific adjusted odds ratio, 95% confidence interval and p-value for each RTD by ethnicity**

| **RTD** |  | **White** | **Black** | | | **Asian** | | | **Mixed** | | | **Other** | | |
| --- | --- | --- | --- | --- | --- | --- | --- | --- | --- | --- | --- | --- | --- | --- |
|  |  | Baseline | AOR | 95% CI | p-value | AOR | 95% CI | p-value | AOR | 95% CI | p-value | AOR | 95% CI | p-value |
| **Emergency** | Breast |  | 0.67 | 0.44-1.01 | 0.05 | 0.59 | 0.40-0.87 | <0.01 | 0.75 | 0.68-0.82 | <0.0001 | 2.13 | 1.63-2.79 | <0.0001 |
|  | Lung |  | 0.88 | 0.73-1.07 | 0.20 | 0.81 | 0.68-0.96 | 0.02 | 0.86 | 0.82-0.90 | <0.0001 | 1.37 | 1.21-1.56 | <0.0001 |
|  | Prostate |  | 0.72 | 0.60-0.87 | <0.001 | 0.69 | 0.52-0.92 | 0.01 | 0.82 | 0.76-0.88 | <0.0001 | 1.62 | 1.25-2.09 | <0.001 |
|  | Colorectal |  | 0.73 | 0.60-0.89 | <0.001 | 0.65 | 0.53-0.79 | <0.0001 | 0.81 | 0.77-0.85 | <0.0001 | 1.55 | 1.34-1.81 | <0.0001 |
|  | Oesophagus |  | 1.01 | 0.58-1.77 | 0.96 | 0.96 | 0.60-1.54 | 0.87 | 0.84 | 0.75-0.94 | <0.01 | 1.28 | 0.93-1.77 | 0.13 |
|  | Stomach |  | 0.99 | 0.73-1.34 | 0.96 | 1.34 | 0.96-1.87 | 0.09 | 0.94 | 0.84-1.04 | 0.23 | 1.48 | 1.11-1.97 | <0.01 |
|  | Cervix |  | 1.05 | 0.48-2.26 | 0.91 | 0.55 | 0.19-1.57 | 0.26 | 0.71 | 0.52-0.97 | 0.03 | 2.19 | 0.97-4.95 | 0.06 |
|  | Myeloma |  | 0.53 | 0.40-0.71 | <0.0001 | 0.77 | 0.54-1.09 | 0.14 | 0.91 | 0.81-1.03 | 0.12 | 1.04 | 0.67-1.61 | 0.88 |
|  | Ovary |  | 1.49 | 0.92-2.41 | 0.10 | 0.95 | 0.68-1.32 | 0.74 | 0.94 | 0.84-1.05 | 0.31 | 1.56 | 1.09-2.22 | 0.01 |
|  | Oral |  | 1.45 | 0.76-2.78 | 0.26 | 0.33 | 0.16-0.71 | <0.01 | 0.81 | 0.67-0.98 | 0.03 | 1.36 | 0.65-2.86 | 0.41 |
| **Elective GP** | Breast |  | 1.52 | 1.27-1.82 | <0.0001 | 1.24 | 1.05-1.45 | 0.01 | 0.95 | 0.89-1.01 | 0.07 | 1.46 | 1.18-1.80 | <0.001 |
|  | Lung |  | 1.01 | 0.81-1.25 | 0.93 | 1.59 | 1.34-1.90 | <0.0001 | 1.05 | 1.00-1.10 | 0.05 | 0.77 | 0.77-0.65 | <0.01 |
|  | Prostate |  | 1.08 | 0.98-1.25 | 0.12 | 1.44 | 1.25-1.66 | <0.0001 | 1.02 | 0.98-1.06 | 0.25 | 0.72 | 0.60-0.87 | <0.001 |
|  | Colorectal |  | 1.24 | 1.03-1.49 | 0.02 | 1.34 | 1.14-1.58 | <0.001 | 1.07 | 1.02-1.12 | <0.01 | 0.79 | 0.66-0.95 | 0.01 |
|  | Oesophagus |  | 1.16 | 0.65-2.08 | 0.61 | 1.72 | 1.13-2.61 | 0.01 | 1 | 0.89-1.12 | 0.96 | 0.81 | 0.55-1.19 | 0.28 |
|  | Stomach |  | 0.89 | 0.63-1.25 | 0.51 | 0.98 | 0.67-1.43 | 0.92 | 0.98 | 0.87-1.11 | 0.77 | 0.74 | 0.52-1.06 | 0.10 |
|  | Cervix |  | 2.38 | 1.33-4.26 | <0.01 | 1.78 | 1.01-3.13 | 0.05 | 0.91 | 0.73-1.14 | 0.41 | 1.01 | 0.48-2.12 | 0.99 |
|  | Myeloma |  | 1.25 | 0.96-1.63 | 0.09 | 1.18 | 0.85-1.65 | 0.33 | 1.11 | 0.98-1.25 | 0.08 | 0.65 | 0.40-1.07 | 0.09 |
|  | Ovary |  | 0.98 | 0.58-1.64 | 0.94 | 0.99 | 0.72-1.37 | 0.96 | 0.95 | 0.84-1.07 | 0.42 | 1.07 | 0.72-1.60 | 0.73 |
|  | Oral |  | 1.4 | 0.91-2.14 | 0.12 | 1.35 | 1.04-1.76 | 0.02 | 1.06 | 0.96-1.18 | 0.27 | 0.95 | 0.58-1.55 | 0.83 |

| **TWW** | Breast |  | 1.18 | 1.03-1.36 | 0.02 | 1.09 | 0.97-1.22 | 0.15 | 0.99 | 0.95-1.03 | 0.58 | 0.68 | 0.58-0.79 | <0.0001 |
| --- | --- | --- | --- | --- | --- | --- | --- | --- | --- | --- | --- | --- | --- | --- |
|  | Lung |  | 0.9 | 0.72-1.11 | 0.32 | 0.69 | 0.56-0.85 | <0.001 | 1.09 | 1.04-1.13 | <0.001 | 0.79 | 0.68-0.91 | <0.01 |
|  | Prostate |  | 1.16 | 1.05-1.28 | <0.01 | 0.82 | 0.71-0.96 | 0.01 | 1.02 | 0.99-1.06 | 0.23 | 0.89 | 0.75-1.06 | 0.19 |
|  | Colorectal |  | 1.03 | 0.86-1.24 | 0.74 | 0.85 | 0.72-1.01 | 0.06 | 1.08 | 1.03-1.12 | <0.001 | 0.76 | 0.65-0.90 | 0.001 |
|  | Oesophagus |  | 0.59 | 0.35-1.00 | 0.05 | 0.54 | 0.35-0.84 | <0.01 | 1.05 | 0.96-1.15 | 0.28 | 0.94 | 0.71-1.25 | 0.68 |
|  | Stomach |  | 0.98 | 0.71-1.35 | 0.91 | 0.69 | 0.46-1.03 | 0.06 | 0.96 | 0.86-1.07 | 0.49 | 0.77 | 0.56-1.07 | 0.12 |
|  | Cervix |  | 0.45 | 0.20-1.04 | 0.06 | 0.83 | 0.41-1.66 | 0.59 | 1.09 | 0.87-1.36 | 0.47 | 0.57 | 0.23-1.42 | 0.23 |
|  | Myeloma |  | 1.55 | 1.13-2.12 | <0.01 | 1.11 | 0.73-1.70 | 0.62 | 1.1 | 0.95-1.28 | 0.19 | 0.77 | 0.42-1.44 | 0.42 |
|  | Ovary |  | 0.67 | 0.38-1.20 | 0.19 | 1.13 | 0.83-1.55 | 0.44 | 1.11 | 1.00-1.24 | 0.06 | 0.43 | 0.27-0.69 | <0.001 |
|  | Oral |  | 0.39 | 0.24-0.63 | <0.001 | 0.58 | 0.44-0.77 | <0.0001 | 0.99 | 0.90-1.09 | 0.87 | 0.82 | 0.53-1.28 | 0.38 |
| **Screening** | Breast |  | 0.77 | 0.64-0.92 | <0.01 | 0.95 | 0.83-1.09 | 0.45 | 1.11 | 1.06-1.16 | <0.0001 | 0.69 | 0.56-0.83 | <0.001 |
|  | Colorectal |  | 1.08 | 0.75-1.57 | 0.68 | 1.67 | 1.29-2.16 | <0.001 | 1.07 | 0.99-1.16 | 0.09 | 0.43 | 0.28-0.63 | <0.0001 |
|  | Cervix |  | 0.66 | 0.26-1.64 | 0.37 | 0.87 | 0.42-1.81 | 0.71 | 1.24 | 0.95-1.60 | 0.11 | 0.67 | 0.25-1.84 | 0.44 |
| **Hospital** | Breast |  | 1.17 | 0.80-1.70 | 0.42 | 1.05 | 0.76-1.45 | 0.76 | 0.96 | 0.86-1.08 | 0.51 | 1.03 | 0.67-1.60 | 0.89 |
|  | Lung |  | 1.46 | 1.15-1.86 | <0.01 | 1.12 | 0.88-1.42 | 0.36 | 1.06 | 1.00-1.12 | 0.06 | 0.76 | 0.61-0.93 | 0.01 |
|  | Prostate |  | 0.76 | 0.64-0.90 | <0.01 | 0.75 | 0.58-0.96 | 0.02 | 1 | 0.94-1.06 | 0.9 | 0.87 | 0.66-1.14 | 0.31 |
|  | Colorectal |  | 1.05 | 0.81-1.35 | 0.73 | 1.11 | 0.89-1.39 | 0.34 | 1.02 | 0.96-1.09 | 0.5 | 1.02 | 0.82-1.27 | 0.88 |
|  | Oesophagus |  | 1.44 | 0.79-2.61 | 0.23 | 1.18 | 0.72-1.94 | 0.51 | 1.11 | 0.99-1.24 | 0.08 | 0.94 | 0.64-1.37 | 0.74 |
|  | Stomach |  | 1 | 0.68-1.48 | 0.99 | 0.95 | 0.61-1.47 | 0.81 | 1.16 | 1.02-1.32 | 0.03 | 0.62 | 0.39-0.97 | 0.03 |
|  | Cervix |  | 0.76 | 0.23-2.52 | 0.66 | 0.94 | 0.33-2.69 | 0.92 | 1.21 | 0.86-1.72 | 0.27 | 1.51 | 0.52-4.40 | 0.45 |
|  | Myeloma |  | 1.07 | 0.73-1.57 | 0.73 | 1 | 0.61-1.64 | 1 | 0.89 | 0.74-1.07 | 0.2 | 1.31 | 0.72-2.39 | 0.37 |
|  | Ovary |  | 0.88 | 0.43-1.79 | 0.73 | 0.67 | 0.41-1.09 | 0.11 | 0.95 | 0.82-1.10 | 0.49 | 0.75 | 0.43-1.31 | 0.31 |
|  | Oral |  | 1.44 | 0.89-2.35 | 0.14 | 1.58 | 1.19-2.11 | <0.01 | 1.01 | 0.90-1.14 | 0.88 | 1.2 | 0.71-2.01 | 0.49 |

| **Other** | Breast |  | 0.57 | 0.37-0.89 | 0.01 | 1.04 | 0.79-1.36 | 0.79 | 1.04 | 0.95-1.14 | 0.43 | 2.5 | 1.95-3.20 | <0.0001 |
| --- | --- | --- | --- | --- | --- | --- | --- | --- | --- | --- | --- | --- | --- | --- |
|  | Lung |  | 0.62 | 0.32-1.21 | 0.16 | 1 | 0.61-1.63 | 0.99 | 1.02 | 0.90-1.16 | 0.72 | 2.61 | 2.04-3.34 | <0.0001 |
|  | Prostate |  | 0.84 | 0.62-1.14 | 0.26 | 1.09 | 0.76-1.58 | 0.63 | 1.14 | 1.03-1.25 | 0.01 | 2.93 | 2.24-3.84 | <0.0001 |
|  | Colorectal |  | 0.83 | 0.48-1.42 | 0.49 | 0.92 | 0.59-1.44 | 0.72 | 1.09 | 0.96-1.22 | 0.18 | 2.48 | 1.88-3.27 | <0.0001 |
|  | Oesophagus |  | 2.57 | 0.91-7.28 | 0.08 | 1.49 | 0.54-4.13 | 0.44 | 0.95 | 0.72-1.26 | 0.74 | 1.4 | 0.70-2.79 | 0.34 |
|  | Stomach |  | 1.6 | 0.79-3.25 | 0.18 | 1.21 | 0.51-2.82 | 0.66 | 1.07 | 0.79-1.45 | 0.64 | 3.66 | 2.27-5.90 | <0.0001 |
|  | Cervix |  | 0.57 | 0.37-0.89 | 0.01 | 0.56 | 0.07-4.18 | 0.57 | 0.72 | 0.37-1.39 | 0.33 | 0.7 | 0.09-5.36 | 0.73 |
|  | Myeloma |  | 0.56 | 0.22-1.43 | 0.23 | 0.74 | 0.27-2.08 | 0.57 | 0.71 | 0.47-1.07 | 0.1 | 4.46 | 2.21-8.98 | <0.0001 |
|  | Ovary |  | 0.44 | 0.06-3.20 | 0.42 | 1.53 | 0.81-2.91 | 0.19 | 1.12 | 0.86-1.45 | 0.42 | 2.62 | 1.47-4.67 | <0.01 |
|  | Oral |  | 3.66 | 1.61-8.34 | 0.01 | 2.32 | 1.32-4.05 | 0.003 | 1.06 | 0.79-1.42 | 0.69 | 1.25 | 0.38-4.08 | 0.71 |

Adjusted for age, sex, IMD, morbidity score, cancer sites and region (including two-way interactions between cancer and age, sex, IMD, and ethnicity)
